# Genome-Wide Patterns of Genetic Variation within and among Alternative Selective Regimes

**DOI:** 10.1371/journal.pgen.1004527

**Published:** 2014-08-07

**Authors:** Yuheng Huang, Stephen I. Wright, Aneil F. Agrawal

**Affiliations:** Department of Ecology & Evolutionary Biology, University of Toronto, Toronto, Ontario, Canada; University of California Davis, United States of America

## Abstract

Environmental heterogeneity has been hypothesized to influence levels of genetic variation but the effect of heterogeneity depends on (i) the form of heterogeneity, (ii) whether ecologically relevant or neutral loci are being considered, and (iii) the genetic basis of ecological adaptation. We surveyed genome-wide SNP diversity in replicate experimental *Drosophila melanogaster* populations with equal census sizes that evolved for 42 generations under one of four selection regimes: (i) salt-enriched environment (*Salt*), (ii) cadmium-enriched environment (*Cad*), (iii) temporally (*Temp*) or (iv) spatially (*Spatial*) variable environments. There was significant differentiation between all pairs of treatments but the greatest differentiation occurred between the two homogenous treatments (*Cad* and *Salt*). For sites likely under differential ecological selection (and those closely linked to them), the pattern of within-population diversity π followed the expectation from classic antagonistic selection theory: *Spatial*>*Temp*>*Salt*≈*Cad*. However, neutral diversity unlinked to selected sites followed a different pattern: *Spatial*>*Salt*≈*Cad*>*Temp*. As implicated by the latter result, measures of *F_ST_* among replicate populations within treatments are consistent with differences in effective population sizes among selective regimes despite equal census sizes. Though there are clear changes in the rank order of treatments when contrasting selected and neutral sites with respect to π, the rank ordering of treatments with respect to *F_ST_* appears reasonably consistent between site categories. These results demonstrate that alternative selective regimes affect within- and among-population diversity differently for different site types.

## Author Summary

Evolutionary biologists seek to understand the factors affecting genetic variation. While it is intuitive that environmental heterogeneity should increase levels of variation, theoretical models showed that spatial and temporal heterogeneity differ in how likely they are to maintain polymorphisms affecting fitness. We evolved experimental populations of fruit flies in constant environments or in temporally or spatially varying environments, then examined levels of sequence variation across the genome. For sites associated with ecological selection, polymorphism patterns matched the theoretical expectations with variation greatest in populations evolving in spatially heterogeneous environments, less variation in populations evolving in temporally heterogeneous environments, and least variation in populations evolving in constant environments. However, a different pattern was observed at sites not associated with differential ecological selection (i.e., most of the genome). For these sites, levels of variation were highest at spatially heterogeneous populations but lowest for temporally heterogeneous populations. Populations evolving under temporal heterogeneity also showed the greatest differentiation from one another, suggesting that this selection regime caused more genetic drift than other selection regimes. These results illustrate that environmental heterogeneity affects levels of variation not only at sites subject to differential ecological selection but also genome-wide, though spatial and temporal heterogeneity affect diversity differently.

## Introduction

Patterns of genetic variation have several major consequences for evolution. First, the amount and type of genetic variation in fitness mediate a population's ability to adapt to selection pressures. Second, the nature of genetic variation determines the fitness consequences of different types of reproduction (e.g., sexual versus asexual reproduction, inbreeding versus outbreeding, random mating versus mate choice), and can therefore be a major cause of selection on reproductive traits. Third, the amount of neutral variation determines the rate at which populations diverge by drift. But what factors shape variation within populations and create differences in levels of variation among them? This is one of the central questions in evolutionary biology [1], [2]. Here we examine how different selective regimes alter patterns of variation across the genome.

When considering genetic variation in fitness, selection must be a major determinant, but the relative importance of different types of selection is unknown. One possibility is that most genetic variation in fitness is due to the constant input of new deleterious mutations balanced by the removal of such variants by negative selection. While mutation-selection balance undoubtedly contributes to variation in fitness, several authors have argued that this model is unable to fully account for empirical observations [3], [4]. The alternative is that some form of balancing selection (e.g., negative frequency-dependent selection, antagonistic pleiotropy, environmental heterogeneity) actively maintains multiple alleles at selected sites.

In theoretical models, the form of selection is imposed by assumption, but, in nature, selection is a consequence of the environment. A common feature of natural environments is that they change over time and space. Here we use experimental evolution to ask how this key feature affects genome-wide patterns of selected and neutral variation. Below we review several simplistic predictions for selected sites but emphasize that these are based on alternative assumptions regarding how environmental heterogeneity changes selection and ignore the effects of linked loci.

Classic theory [5], [6] predicts that genetic variation at selected sites can be maintained by environmental heterogeneity if alternative alleles are favoured in different environments (environmentally antagonistic selection, EAS). However, the type of heterogeneity is important; the conditions for a protected polymorphism through balancing selection are more restrictive with temporal heterogeneity than spatial heterogeneity [6], [7]. From this, we expect to find the most genetic variation for fitness in spatially heterogeneous populations, somewhat less in temporally heterogeneous populations, and the least variation in populations evolving in a constant environment (i.e., *V_Spatial_*>*V_Temp_*>*V_Const_*).

Rather than being environmentally antagonistic, allelic variation could be conditionally neutral (CN) whereby the two alleles at a site are selectively neutral in Environment A but with differential fitness effects in Environment B. The disfavored allele will be purged in Environment B but the polymorphism could persist for much longer in Environment A. For populations that experience both environments, conditionally neutral alleles experience half as much directional selection. Therefore, the heterogeneous regimes are expected to harbor levels of genetic variation for this locus intermediate to that in populations maintained in A or B alone (i.e., *V_Const_A_*>*V_Spatial_*>*V_Temp_*>*V_Const_B_*). However, if we consider multiple loci and assume that some experience selection only in A and others only in B, then the average amount of variation across these loci might be lowest in the heterogeneous populations where variation should be purged eventually from both types of loci (i.e., *V_Const_A_*, *V_Const_B_*>*V_Spatial_*>*V_Temp_*).

Rather than favoring the phenotypes specialized for either A or B, a third possibility is that heterogeneous environments select for a distinct phenotype (e.g., a robust generalist rather than the maintenance of multiple specialists). In this case, populations from heterogeneous environments would not be genetically intermediate between populations from alternative constant environments; rather, they might be as genetically distinct from the homogenously selected populations as the homogenously selected populations are from one another. Under this scenario, there is no reason to expect levels of variation to differ among selective regimes.

While some sites experience different selection between different environments or between homogeneous versus heterogeneous regimes, most sites will be uniformly selected across environments or neutral in both environments. However, variation at such sites will be influenced by changes in selection at other sites in the genome due to genetic hitchhiking (linkage effect). The extent of this influence will depend on the number of differentially selected sites, the strength, form and timescale of selection on these other sites, as well as their distribution across the genome and the recombination rates between them [8]–[10].

A number of important experimental studies have examined the effect of environmental heterogeneity on genetic variance (quantitative genetic variation: [11]–[17]; allozyme heterozygosity: [18]–[20]). The findings from these studies are quite variable and often there are few significant differences between treatments. Logistical constraints have limited past studies to either quantitative genetic analysis of a small number of traits or diversity measures from a handful molecular markers. It is impossible to know whether unmeasured traits or loci were similarly affected. An additional challenge in interpreting past results is that it is often unclear whether the traits or loci being studied are under differential selection between environments or even closely related to fitness.

High-throughput re-sequencing offers new opportunities to re-examine this issue. This technique has been applied to study the genetic basis of selection response in experimental populations (e.g., [21]–[24]). We applied this technology to replicated experimental populations of *Drosophila melanogaster* that evolved under different selective regimes. Twenty replicate populations were established from a cross between an ancestral lab population adapted to a salt-enriched larval environment (“*Ancestral Salt*,” *AS*) and an ancestral lab population adapted to a cadmium-enriched larval environment (“*Ancestral Cad*,” *AC*). These 20 replicates (*N* = 448 per population) were divided equally among four treatments: two constant treatments in either salt- (*Salt*) or cadmium-enriched (*Cad*) larval environments, a temporally heterogeneous treatment (*Temp*) in which populations were switched between each generation to the alternate environment, and a spatially heterogeneous treatment (*Spatial*) in which the population was split between the two environments but surviving adults were mixed with equal number from the two environments before egg-laying for next generation. After ∼20 generations, Long et al [25] found striking differences across treatments in inbreeding depression, suggesting important shifts in patterns of variation within populations among treatments.

**Figure 1:**
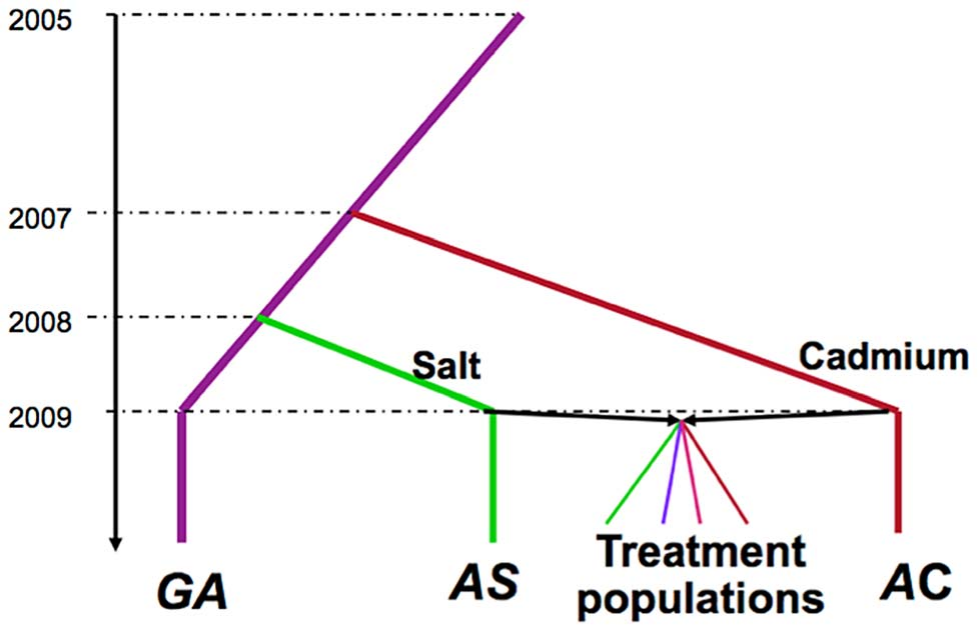
Selection history of the experimental populations. The Grand Ancestor population (*GA*) was maintained in benign laboratory conditions, and was used to initiate populations maintained on salt-enriched media (*AS*) or cadmium-enriched media (*AC*). The treatment populations were produced by crossing two ancestral population *AS* and *AC*. There are 5 replicate populations of each of the four treatments (not illustrated).

At generation 42, we performed pooled re-sequencing of each of the 20 populations, the two environmentally specialized ancestral populations (*AS* and *AC*), as well as the lab source population (“*Grand Ancestor*”, *GA*) from which *AS* and *AC* were derived (see Figure 1 for relationship among populations). We first examine genetic differentiation among treatments and identify candidate sites under differential selection. We then compare levels of within-population variation among treatments. There are major differences in levels of variation among treatments and these patterns change between neutral and putatively selected sites.

## Results

### Differentiation for environment-specific survival

**Figure 2:**
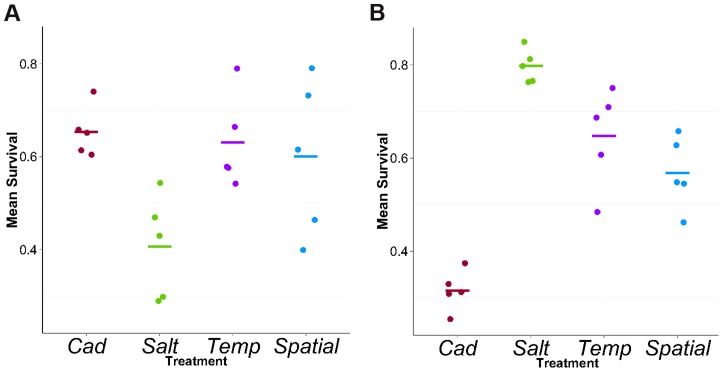
Mean survival in cadmium (A) or salt (B) environment. Each point represents the mean survival of ∼140 full-sib families for each population. Each bar represents the mean across five replicate populations for each treatment.

We tested the survival of flies from all replicate populations in both environments. Contrasting the two constant treatments, we found the expected patterns of local adaptation, i.e., *Cad* populations had higher survival than *Salt* populations when tested in cadmium but the reverse was true in salt (Figure 2). In the salt (cadmium) environment, the survival rates of flies from heterogeneous treatments were considerably higher than flies from *Cad* (*Salt*) treatment, indicating populations perform poorly when tested in an environment to which they lack selective exposure. These results are qualitatively similar to those reported by Long et al [25] from an earlier time point (∼generation 20).

### Allele frequency differentiation between salt and cadmium environments

In addition to measuring the differentiation in survival, we measured differentiation in the frequencies of single nucleotide polymorphisms (SNPs) between the *Salt* treatment and *Cad* treatment. After read mapping and filtering for map and base quality, we had a mean coverage among populations of ∼17.5 fold for euchromatic regions.

To maximize our power in examining differentiation that occurs between populations evolving in the two extreme environments, we compared allele frequencies in the Ancestral Salt (*AS*) and the five *Salt* populations with allele frequencies in the Ancestral Cadmium (*AC*) and the five *Cad* populations. To identify SNPs, we screened for sites in euchromatic regions that are ≥5-fold coverage in all of these populations. We kept only those sites where the initial diversity (π*_ini_*) was not too low, specifically π*_ini_* = 2p*_ini_* *(1−p*_ini_*)>5%, where p*_ini_* is the estimated frequency of the minor allele pooling across the Ancestral Salt (*AS*) and Ancestral Cad (*AC*) populations. After applying these screens, there were ∼2*10^6^ sites for testing differentiation; we refer to these sites as the “α-sites”. To maximize our statistical power, we considered allele frequency estimates from the five replicate *Salt* populations and the Ancestral Salt (*AS*) population as replicates for a “salt” treatment and the estimates from the five replicate *Cad* populations and the Ancestral Cad (*AC*) as replicates for a “cadmium” treatment and then used a screen for allele frequency differentiation between treatments based on the Cochran-Mantel-Haenszel test (CMH) [26]. A total of 123,291 sites passed our screen for significant differentiation with false discovery rate = 0.001%; we refer to these as the “β-sites”. Because differentiation likely occurs from common variants, we expect the variation in the Grand Ancestor population to be greater at β-sites than other α-sites. Indeed, diversity is ∼20% greater (i.e., π*_GA_*_, β-sites_ = 0.290; π*_GA_*_, other α-sites_ = 0.246).

To evaluate the potential for false positives due to sampling error, allele frequency estimation error or genetic drift, we randomly assigned half of our cadmium populations as well as half of our salt populations to pseudo-treatment “A” and the remaining cadmium and salt populations to pseudo-treatment “B”. We then performed the same analysis looking for differentiation between “A” and “B”. We repeated this process for 15 randomly chosen combinations. Among these combinations, we found the mean number of sites that passed the same criteria above was 93, with the highest number 349. Thus, we expect the majority of the ∼123000 significant differentiated sites are not false positives in a statistical sense.

**Figure 3:**
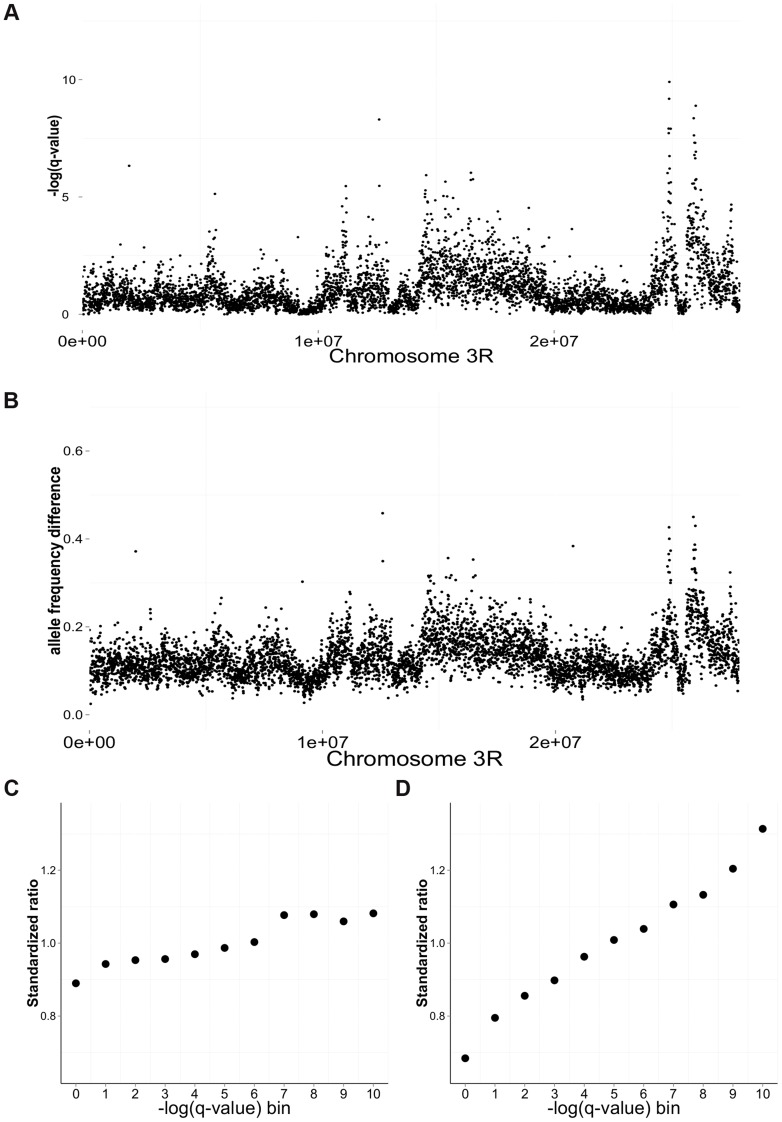
Differentiation between salt and cadmium populations. Sliding-window (5 kb non-overlapping) plots of differentiation along chromosome 3R: the average -log(q-value) from the CMH test (A) and the average difference in mean allele frequency (B) between the salt and cadmium populations. For each environment, the data are calculated based on the environment-specific ancestor and the five replicate populations. The ratio of the number of genic to intergenic sites (C) and the ratio of the number of coding sites to intronic sites (D) in different -log(q-value) bins using data from the whole genome. To compare the magnitude of the ratios for different functional catergories, the ratios are standardized around 1 by dividing the mean ratio among the 11 bins. Strong significance corresponds to larger values of -log(q-value).

Nonetheless, it is highly improbable that ∼123000 β-sites are direct targets of differential selection. Much of the differentiation is likely the result of linkage effects. In the most extreme situation, there could be just one or two selected sites per chromosome and all the other sites could differentiate because of linkage. However, there are several observations that would not be expected if linkage effects were massive and there were only a few true targets of selection: (1.1) Rather than observing a single large block of differentiation, we observe numerous distinct peaks of differentiation across each chromosome (plotted as non-overlapping sliding windows Figure 3A–B, Supplementary Information S1A and Figure S1 for whole genome); (1.2) Among the most strongly significantly differentiated sites, there is an enrichment of (i) intragenic to intergenic sites, and (ii) coding to non-coding intragenic sites (Figure 3C–D). (There is no enrichment of nonsynonymous sites relative to 4-fold degenerate sites suggesting strong linkage effects at this small scale; more details in Supplementary Information S1B and Figure S2); (1.3) Differentiation is not random with respect to gene function, rather certain classes of genes (Gene Ontology) show enrichment, including those involved with protein metabolic process and peptide activity (Supplementary Information S1C and Table S1). Moreover, similar functional categories are identified when gene enrichment tests are performed separately for the two major autosomes (Table S1). In addition, some of our differentiated genes had been identified in previous functional studies of cadmium and salt resistance (e.g., MtnB, MTF-1 for cadmium resistance [27], [28] and CG6484, sug, Sik3, CrebA, Crtc for salt resistance [29], [30]).

Other patterns suggest that linkage effects are contributing to differentiation but on a scale much smaller than the whole chromosomes: (2.1) The β-sites are strongly (and significantly) clustered relative to the distribution of α sites across genome. However, the level of clustering declines rapidly within 5000 bp, though a lower level of clustering extends much further (Supplementary Information S2A and Figure S3). The pattern of clustering remains similar after controlling for initial diversity in the *GA* population, which could affect the likelihood of differentiation and the power to detect it (not shown); (2.2) *F_ST_* between salt and cadmium populations is very high near focal differentiated sites but declines rapidly within the first 500–1000 base pairs (Supplementary Information S2B and Figure S4); (2.3) The linkage disequilibrium (LD) within the paired-end reads (2*100 bp) declines relatively rapidly within the first 100 bp from 0.7 to about 0.4 (Supplementary Information S2C and Figure S5). These patterns remain similar when we only consider the regions that contain β-sites.

To assay the potential impact of inversions on genetic differentiation and as a source of linkage disequilibria, we used previously identified inversion-specific SNP markers [31] to estimate the inversion frequency in our populations (Supplementary Information S2D). The frequency of all potential inversions across the six populations in constant cadmium and six populations in constant salt environment are lower than 5%. Given the average allele frequency differentiation of β-sites is ∼0.4 (Tables S2, S3 and Supplementary Information S3), inversions are unlikely to play a major role. Further, for the two major autosomes, on average, the relative abundance of the β-sites and level of LD do not seem to be elevated in regions of commonly segregating inversions (Tables S4, S5 and Supplementary Information S5). As the linked selection is expected to be stronger in low recombination regions, we calculated the number and proportion of β-sites and α-sites in each of these regions (Table S6). Consistent with the prediction of greater effects of linked selection, there is a higher proportion of significant sites to α-sites in low recombination regions than high recombination regions (7.6% vs 5.4%, the difference is caused by autosome 2).

Recent papers [32]–[34] suggest that “evolve & re-sequence” studies have limited power to identify true targets of selection and our observation of clustering of β-sites and LD is consistent with those concerns. However, for our purposes, it is not essential to identify the true targets, rather our purpose is to demonstrate that the two alternative habitats cause genetic differentiation. Nonetheless, such differentiation is more meaningful if it is not due to selection on only one or two sites. Taken together, the results above suggest that the β-sites are enriched for true targets of selection but that many of the β-sites are differentiated because of linked selection. Linkage effects are likely strong but the patterns reported above (e.g., enrichment of significant sites in coding regions as well as gene ontology enrichment) do not seem consistent with selection on only a few sites. However, we emphasize that we cannot identify true targets of selection or reliably estimate whether the true number of targets is in the tens, hundreds, or more. We conclude only that the salt and cadmium environments cause populations to diverge genetically and that, though much of the differentiation may be due to linkage effects, there are likely multiple targets of selection.

### Pairwise genetic differentiation between selective regimes

After establishing the high degree of differentiation across the genome between the two constant environments, we examined the level of differentiation between all other pairs of treatments. In the preceding section we compared the salt and cadmium environments and included the ancestral populations to maximize power for finding β-sites. In this section, we use only the five replicate populations within each experimental selection regime (the ancestral populations are not considered) so that we have similar power to examine differentiation between all pairs of treatments (e.g., *Salt* vs. *Cad*; *Temp* vs. *Spatial*). The number of significantly differentiated sites between each pair of treatments is shown in Table 1. The number of differentiated sites between the two homogenous treatments (*Cad* and *Salt*) is much greater than the numbers between any pair of homogeneous and heterogeneous treatments (e.g., *Cad* and *Spatial*). The two heterogeneous treatments (*Spatial* and *Temp*), where populations experience both selective agents, have many fewer differentiated sites than any other pair of treatments. The numbers of genes with significantly differentiated sites shows the same relationship among treatment pairs (Table S7).

**Table 1:** The number of highly differentiated sites between each pair of treatments.

	*Cad*	*Temp*	*Spatial*
*Salt*	90661	9427	6456
*Cad*		14528	3528
*Temp*			488

For each pair of treatments, we performed CMH tests on each site using the five replicate populations from each treatment. For the *Cad* vs *Salt* comparison here we used only the five replicate populations for each treatment; for the main analysis discussed in the text we also included the ancestral populations of each treatment (*AC* and *AS*). We did five different ways of pairings for the tests. The q-value was calculated from the p-value for each pairing in the CMH test and then transformed by the “BY” method. Then we used q-value cutoff equals 10^−5^ to pick up the significant sites that pass this cutoff for all five ways of pairings. For the Salt-Cad pairs in the Table, 93.8% of the significant sites (90661) are found in the β-sites (123291) and for *Temp-Spatial*, it is just 13.7% of them (488). For *Salt-Temp*, *Salt-Spatial*, *Cad-Temp*, *Cad-Spatial* pairs, there are 71.8%, 82.8%, 64.3% and 78.2% of their own significant sites found in the β-sites (six *Salt-*six *Cad*) respectively.

As an alternative approach to comparing treatments, we measured the correlation of allele frequencies between different pairs of treatments using the sites that are likely under differential selection. To avoid bias in comparing treatments, we identified sites that were significantly differentiated in allele frequencies between *AS* and *AC*, the direct ancestors of all treatments (using Fisher's exact test with q-value<0.001). Because a correlation in allele frequency will arise simply from variance among sites in initial allele frequency, we calculated a ‘standardized’ correlation of allele frequency between each treatment pairs by using the correlation for differentially selected sites minus the correlation for non-differentiated control sites (details in Supplementary Information S3 and Table S8). We found the standardized correlation between the *Salt* and *Cad* treatments is significantly negative, and more negative than the correlation between any other pair of treatments. The negative correlation possibly suggests that the most strongly favored alleles in one environment are the most strongly disfavored in the other environment, indicating the operation of environmentally antagonistic selection (EAS). On the other hand, *Temp* and *Spatial* treatments have the most positive correlation, which is consistent with the differentially selected sites experiencing similar selection pressures between the two heterogeneous treatments (Supplementary Information S3 and Table S8).

### Molecular diversity in alternative selective regimes

**Figure 4:**
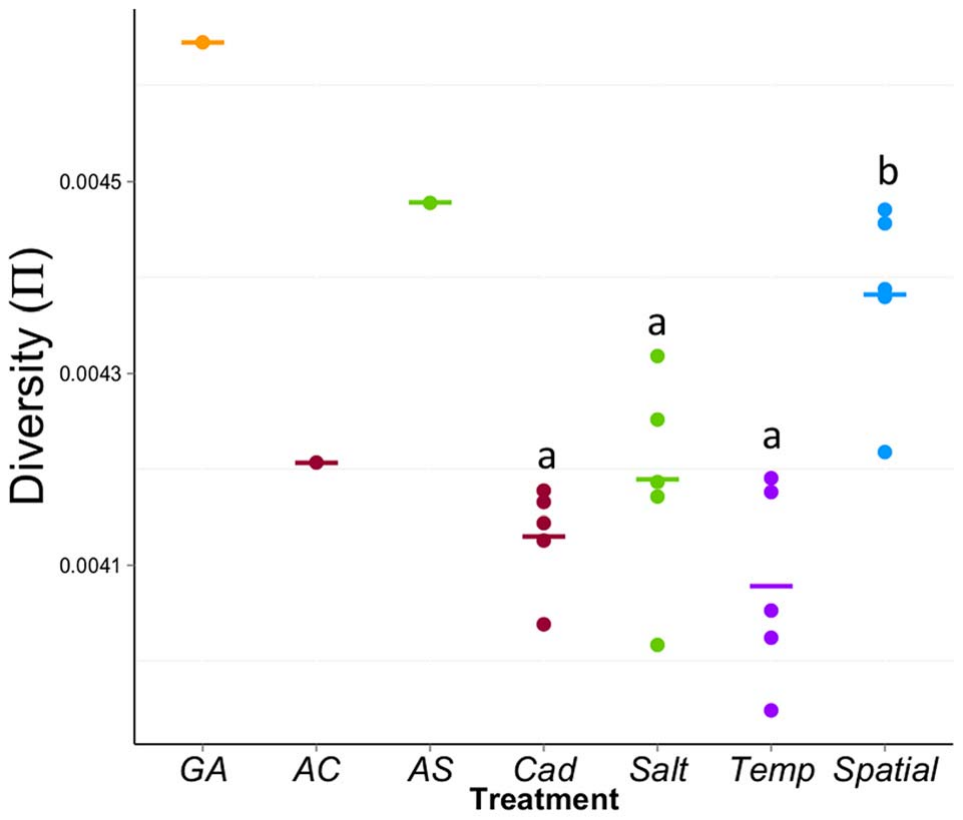
Average diversity (π) within experimental populations across the genome. Each point represents the mean π across the common sliding windows (including non-variable sites) for each population. Though ancestral populations are shown for reference, we are primarily interested in the mean diversity (π) among the four treatments that were created by crossing *AC* and *AS*. There is significant variation among the four treatments (*F*_3,16_ = 9.68, *P* = 0.0007). The “b” group is significantly higher than the “a” group based on ANOVA Tukey HSD test. *Spatial* has significantly higher diversity than the *Temp* (*P*_adj_ = 0.0006, Tukey HSD), the *Salt* (*P*_adj_ = 0.026) and the *Cad* (*P*_adj_ = 0.0036).

Our major goal is to compare the molecular variation among different treatments. First, we compared the genome-wide average diversity (π) among treatments. We used non-overlapping sliding windows to calculate Tajima's π over ∼95% of the euchromatic region of the genome for which we have sufficient coverage in all populations [35], [36]. The mean π across the genome for each population is shown in Figure 4. There is significant variation in π among treatments (*F*_3,16_ = 9.68, *P* = 0.0007); the genome-wide level of diversity is highest for the *Spatial* treatment but lowest for the *Temp* treatment. Though the census size of all populations is equal, given most of the sites across the genome are neutral, the observed differences in genome-wide average π suggest that treatments differ in effective population size. However, the use of genome-wide averages belies more interesting patterns that emerge at a finer scale.

The prediction of *V_Spatial_*>*V_Temp_*>*V_Const_* is for antagonistically selected sites, but many of the variable sites are likely neutral (or effectively neutral). To better test the prediction, we attempted to identify the sites that are likely under differential selection between the salt and cadmium environments. For this section, we want to identify potential targets of differential ecological selection using only the data from the two ancestral populations (*AC* and *AS*). This is necessary to avoid biasing tests comparing among selective treatments. To do so, we applied a different screen than used in the earlier sections. First, to increase the accuracy of the estimation of allele frequency, we screened sites that have ≥15-fold coverage in both ancestral populations and have π*_ini_*>5% (estimated from the average allele frequency in the *AC* and *AS*). Also, we screened for the sites that have ≥10-fold coverage for all of the 20 treatment populations. In total, we had 769,924 SNPs for this analysis; we refer to these as the “χ-sites”.

We calculated the degree of differentiation in allele frequency between the two ancestral source populations *AS* and *AC* for all of the χ-sites, i.e., *d* = |*p_AS_*−*p_AC_*|. Sites under environmentally antagonistic selection will tend to have high *d* values. Because all treatments were created equally from *AS* and *AC*, there should be no initial differences in π among treatments for any given level of *d*. However, differences in π among treatments should develop due to treatment-specific selection, and the nature of these differences is expected to vary with *d* (i.e., selection is likely similar across treatments for low *d* sites whereas high *d* sites likely experience very different selection in different treatments).

**Figure 5:**
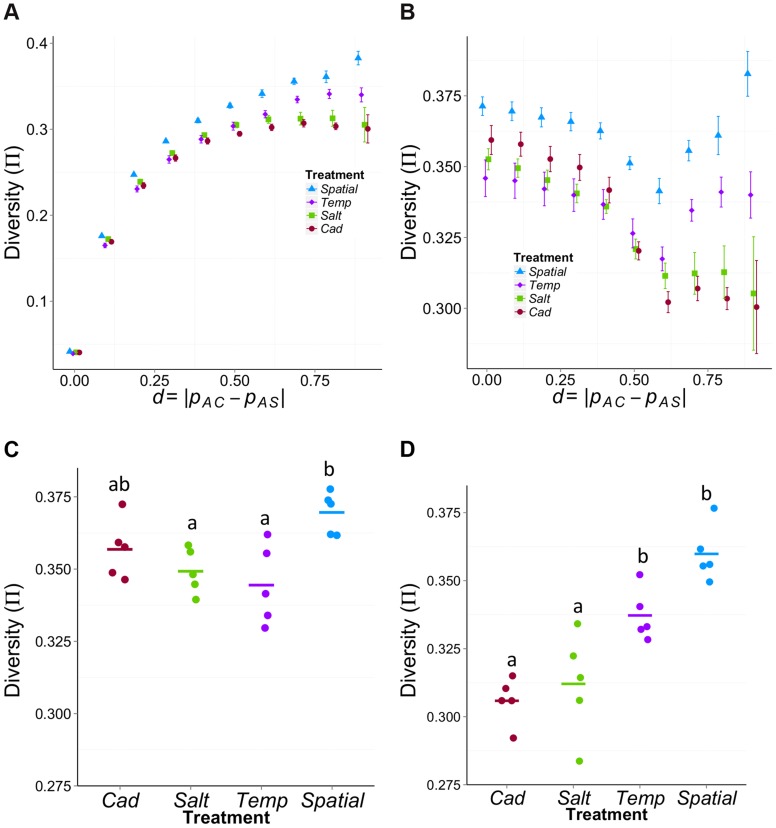
Mean π within treatments as function of differentiation (*d*) between the ancestral source populations (*AC* and *AS*). (A) and (B) The average π across different levels of ancestral differentiation for (A) all χ-site SNPs or (B) only those χ-site SNPs that have high initial diversity (π_ini_>0.4). The *x*-axis is the allele frequency difference between ancestral populations, *d* = |*p_AC_*−*p_AS_*|. Error bars represent the standard error among the five replicates for each treatment. (C) and (D) Comparison among treatments in average π for sites that have high initial diversity (π_ini_>0.4) using sites with (C) weak ancestral differentiation (*d*<0.3) or (D) strong ancestral differentiation (*d*>0.7). For the weakly differentiated sites (C), *Spatial* has significantly higher diversity than the *Temp* (*P*_adj_ = 0.006, Tukey HSD) and the *Salt* (*P*_adj_ = 0.028). For the highly differentiated sites (D), both *Spatial* and *Temp* treatments have significantly higher diversity than the two constant treatments (*P*_adj_<0.03 between any a & b pairs).

To illustrate how the variance changes as we move from neutral sites to sites putatively under differential selection, we calculated π across the complete range of differentiation (*d*) values (Figure 5A). For all treatments, π is lower for weakly differentiated sites than strongly differentiated sites. This pattern is expected simply because the experimental populations were founded by crossing *AC* and *AS*; thus, sites that are strongly differentiated between *AC* and *AS* will start off as highly polymorphic within each experimental population. More interestingly, the rank order of the treatments with respect to diversity changes from weakly differentiated sites (π*_Spatial_*>π*_Salt_*≈π*_Cad_*>π*_Temp_* for *d*<0.3) to highly differentiated ones (π*_Spatial_*>π*_Temp_*>π*_Salt_*≈π*_Cad_* for *d*>0.7).

To more clearly see the effect of selection, it is helpful to compare π between weakly and strongly differentiated sites that have similar levels of initial diversity. To do so, we re-examined the data using only those sites that have high initial diversity (π*_ini_*>0.4, where π*_ini_* = 2*p_ini_*(1−*p_ini_*) and *p_ini_* = ½(*p_AS_+p_AC_*)). Using these sites, the relationship of π to *d* is very different for heterogeneous treatments than for homogeneous treatments (Figure 5B). In the constant selection environments π declines with *d*, as expected under directional selection. In contrast, π is relatively high at both low and high values of *d* for heterogeneous treatments.

When we compare levels of diversity among treatments using the χ-sites with high initial diversity (π*_ini_*>0.4), we find different patterns for sites that are weakly or strongly differentiated between the ancestral founding populations. For the putatively ‘neutral’ sites (arbitrarily designated as *d*<0.3), we find significant variation in π among treatments (*F*_3,16_ = 5.8, *P* = 0.007; Figure 5C) with treatments ranked as π*_Spatial_*>π*_Cad_*>π*_Salt_*>π*_Temp_*. There is also significant variation in π among treatments for the sites likely to be under differential selection (*d*>0.7, *F*_3,16_ = 19.5, *P* = 1.36E^−5^; Figure 5D) but the order is π*_Spatial_*>π*_Temp_*>π*_Salt_*>π*_Cad_*. These differences in diversity are not driven by the potential effect of inversions (which seem to be very rare in our population, Table S2). After excluding the χ-sites located within inversion regions, the diversity patterns are qualitatively similar (Supplementary Information S4 and Figure S6)

### Divergence among replicates within treatments

**Figure 6:**
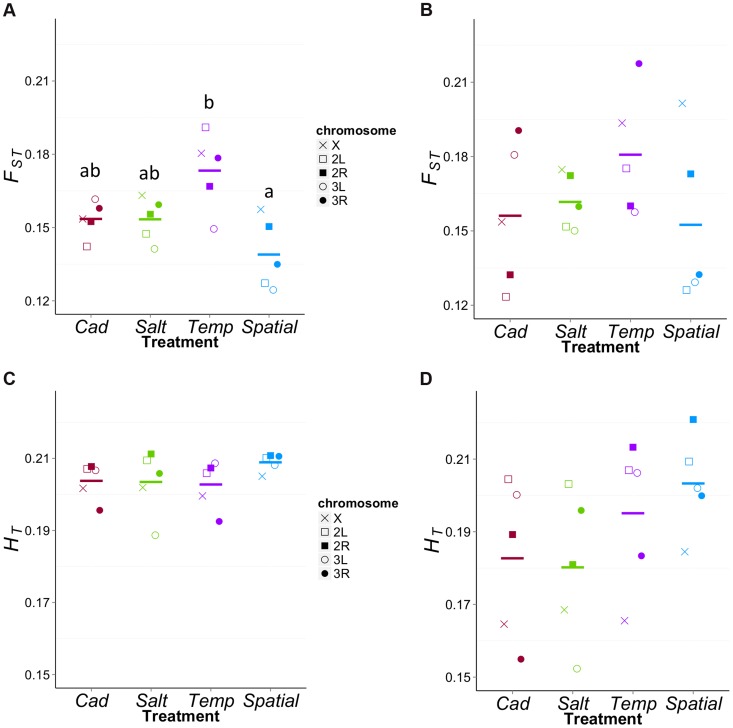
*F_ST_* values for each treatment in different regions. We screened for the χ-sites above with π*_ini_*>0.4 and divided them into five recombinationally independent regions of the genome. For weakly differentiated sites (*d*<0.3, Figure 6A), There is significant variation among treatments in *F_ST_* values with *Temp* being highest among them (*F*_3,16_ = 6.72, *P* = 0.0038). The *Temp* has significantly higher *F_ST_* than *Spatial* (*P*_adj_ = 0.002). The difference between *Temp* and the two constant treatments are marginally non-significant (*Temp* vs. *Salt*: *P*_adj_ = 0.084; *Temp* vs. *Cad*: *P*_adj_ = 0.087). For highly differentiated sites (*d*>0.7, Figure 6B), the *F_ST_* values do not significantly differ among treatments (*F*_3,16_ = 1.16, *P* = 0.35). For both weakly and highly differentiated sites, the treatments do not differ in expected total heterozygosity (*H_T_*) (*F*_3,16_ = 1.02, *P* = 0.41, Figure 6C; *F*_3,16_ = 1.59, *P* = 0.23, Figure 6D).

The reduced level of diversity at ‘neutral’ sites within the *Temp* treatment suggests that populations in this treatment have the lowest *N_e_*. Drift should not only reduce diversity within populations but also cause divergence among populations. To test the latter, we examined *F_ST_* among the five replicate populations within each treatment. For this analysis, we used ‘neutral’ sites passing a minimum coverage and initial diversity thresholds (i.e., the χ-sites above with π*_ini_*>0.4 and *d*<0.3). As expected, *F_ST_* was higher for X chromosome sites than autosomes in all four treatments, (X *F_ST_* = 0.156±007; autosome *F_ST_* = 0.139±008; *t* = −3.87, df = 3, *P* = 0.026). We then compared *F_ST_* among treatments using five regions of the genome that are approximately recombinationally independent: the X chromosome, and the left and right ends of each of the two autosomes (Figure 6A). There was significant variation among treatments in *F_ST_* values (*F*_3,16_ = 6.72, *P* = 0.004), with post-hoc tests showing this was primarily due to elevated *F_ST_* in the *Temp* treatment, consistent with a lower *N_e_* for this treatment. It is worth noting that the treatments do not differ in expected total heterozygosity (*H_T_*) for these sites (*F*_3,16_ = 1.02, *P* = 0.41, Figure 6C), which can be a misleading cause of differences in *F_ST_* [37].

The equivalent comparison of within-treatment *F_ST_* for sites likely to be under differential ecological selection (*d*>0.7) does not show significant differences among treatments (*F*_3,16_ = 1.16, *P* = 0.35, Figure 6B) but the patterns are qualitatively similar to the ‘neutral’ sites. *H_T_* does not vary significantly among treatments (*F*_3,16_ = 1.59, *P* = 0.23; Figure 6D), though point estimates of average *H_T_* are higher in the heterogeneous treatments than the homogeneous ones. It is worth noting that while both heterogeneous treatments have elevated *H_T_* values, the *Temp* treatment has the highest *F_ST_* value whereas the *Spatial* has the lowest. These patterns suggest the differences in *N_e_* between *Spatial* and *Temp* affect differentiation among replicates even at sites under selection (or those linked to them). Based on the low within-population diversity of the constant environments (Figure 5B), we might have expected elevated *F_ST_* for these treatments. However this is not the case and may be attributable to the ‘selected’ sites experiencing stronger net selection in these treatments.

## Discussion

In nature, many populations experience environments that are heterogeneous in space or time. Though our experimental populations differ from natural populations in a number of ways (population size, time scale of adaptation, etc.), our study provides insight into how alternative selective regimes affect genome-wide patterns of molecular diversity in an experimentally simplified setting. In our study, we used two environments that cause populations to diverge with respect to environment-specific survival (Figure 2) as well as allele frequency at numerous SNPs (Figure 3). Though we are unable to determine the number or identity of true genetic targets of differential ecological selection, our analyses suggest that there are likely to be more than one or two per chromosome.

For variants that are likely under differential selection between cadmium and salt environments (or statistically linked to such sites), the diversity patterns are consistent with the classic theoretical prediction: *V_Spatial_*>*V_Temp_*>*V_Const_* (Figure 5D). In contrast, the pattern of diversity of other sites is different: *V_Spatial_*>*V_Const_*>*V_Temp_* (Figures 4 and 5C). In other words, the rank order of treatments with respect to genetic diversity differs between ecologically selected sites and other sites. Our classification of ‘selected’ sites likely includes many neutral sites that are statistically linked to true targets of selection, due to physical linkage or, perhaps more importantly, through admixture or stochastic association from the founding of these populations [32]–[34]. However, for our purposes of studying diversity, such sites behave similarly to sites under differential ecological selection because of their statistical association with true targets.

The difference in the rank order of treatments between ‘selected’ and other sites may help to reconcile inconsistencies among classic studies [11]–[20] attempting to assess how alternative selective regimes affect genetic variance in phenotypes or genetic markers. In most of those studies, phenotypes or genetic markers had unknown relationships to experimentally imposed ecological differences in selection. Even in cases where measured phenotypes or markers are unlikely to have any true ecological effect, they might have been statistically linked to genes that did, making it difficult to interpret such results. By categorizing sites based on information from the ancestral populations, we found very different patterns of variation among our experimental treatments for different categories of sites within the context of a single study.

The difference in the rank order of treatments between ‘selected’ and other sites seems to occur primarily because of how selection affects diversity in homogeneous environments. Focusing on a set of sites that should have similar initial levels of diversity (Figures 5B–D), we found that diversity levels in the two heterogeneous treatments were similar between ‘selected’ and ‘neutral’ sites, but with diversity in *Temp* being consistently ∼6% lower than in the *Spatial* treatment. In contrast, diversity in homogeneous treatments is ∼14% lower than the *Spatial* treatment for ‘selected’ sites but only ∼4% lower for neutral sites.

In simplistic simulations with environmentally antagonistic alleles (Supplementary Information S5 and Figure S7), we were able to qualitatively reproduce these patterns by considering physical linkage to selected sites. In constant environments, neutral diversity was reduced in simulations where the neutral site was closer to selected targets compared to simulations where the recombination distance was greater. For simulated populations evolving in spatially heterogeneous environments, physical linkage to selected sites had only weak effect on neutral diversity, unless selection was strong. With temporal heterogeneity there was no noticeable effect of physical linkage on neutral diversity.

Spatial heterogeneity can result in balancing selection. Previous theoretical studies have shown that balancing selection is expected to increase neutral diversity, but only at sites closely linked to the targets of balancing selection [38]. In contrast, Barton [39] showed that temporally fluctuating selection is expected to reduce neutral variation across the genome (see also [40], [41]). When linkage is loose relative to selection, the drift experienced by neutral sites will be increased by an amount proportional to the enhanced amount of additive genetic variation maintained by fluctuating selection. For more closely linked sites, the loss of diversity can be much more extreme if allele frequencies at the selected loci are oscillating between extreme values. Fluctuating selection is only expected to increase diversity for sites with extremely tight linkage and only when time scales are very long [39]. The latter effect results from the build up of mutations during the longer coalescent times of sites linked to targets of balancing selection relative to those sites that are unlinked. However, if fluctuating selection is applied to standing variation for a short period, as in our simulations and experiment, there appears to be little relationship between physical linkage to selected sites and diversity.

The patterns in the simulations are qualitatively similar to those in our data if we assume our strongly differentiated sites (*d*>0.7; Figure 5D) are more tightly linked to true selected targets than are our ‘neutral’ sites (*d*<0.3). Another approach to examining the importance of linkage is to compare diversity in regions of high versus low recombination. If we assume that neutral sites will tend to be more tightly linked to a true target in the regions of low versus high recombination, then there should be differences in the diversity between low and high recombination regions but these differences should vary among treatments. Controlling for initial diversity of sites among regions, there is significantly less variation in low recombination regions than in high recombination regions in the *Cad* (Supplementary Information S6, Figures S8 and S9, Table S9), as expected. However, diversity does not differ between high and low recombination regions in the other treatments. This lack of a difference is reasonably consistent with the predictions of our simulations (Figure S7) and our analysis based on *d* (Figure 5B) for the two heterogeneous treatments but not for *Salt*. The contrast between high and low recombination regions used here is a crude comparison because (i) the distribution of true selected targets across these regions is unknown and (ii) even the “low” recombination regions likely include many neutral sites that have low degree of linkage from selected sites.

Differential selection between environments could take two qualitatively different, but not mutually exclusive, forms: environmentally antagonistic selection (EAS) and conditional neutrality (CN). This experiment was not designed to distinguish between them but we can consider the observed patterns in light of these alternatives. For sites strongly differentiated between the ancestral populations (*AS* and *AC*), the diversity pattern in the experimental populations is consistent with the prediction from the antagonistic selection model. After controlling for initial diversity, diversity is reduced for putatively selected sites compared to other sites in the constant selection treatments but not in the heterogeneous treatments (Figure 5B). Under CN we would expect the heterogeneous environments to show a decline in diversity for selected sites, albeit a less severe decline than in the constant environments. In contrast, the diversity increases for sites expected to be more strongly selected (i.e., π increases with *d* for *d*>0.6, Figure 5B), suggestive of EAS. An additional observation suggestive of antagonistic selection is the negative correlation in allele frequency between *Salt* and *Cad* treatments (Supplementary Information S3 and Table S8).

Despite these observations consistent with EAS, we suspect that some sites are conditionally neutral. A curious pattern is the clear dip in diversity seen in Figure 5B for both heterogeneous treatments for sites with intermediate levels of divergence between the two ancestral populations (0.4<*d*<0.6). One possible explanation emerges from considering the type of sites likely to be found at different levels of *d* (where *d* = |*p_AC_*−*p_AS_*|). Low values of *d* will be enriched for sites that are neutral or uniformly selected across environments whereas high values of *d* will be enriched for environmentally antagonistically selected sites. Compared to EAS sites, conditionally neutral sites will tend to show less differentiation between the two ancestral populations (i.e., intermediate values of *d*) because they are selected in only one ancestor and are neutral in the other. In the heterogeneous habitats, variation from such sites is expected to be eliminated because one allele is deleterious some of the time and never advantageous. This would explain the reduced diversity for sites with intermediate values of *d* compared to neutral sites (low *d* sites).

Conditionally neutral alleles should show evidence of being selected in one environment but not the other. We cannot rigorously test for this but, as a first approximation, we can consider the fraction of sites that are differentiated between environments but also show little change in allele frequency from the ancestral state in one of the two habitats. Of sites significantly differentiated between salt and cadmium (β-sites), ∼5% (6620/123291) could be classified as possibly neutral in cadmium based on showing low differentiation between the *Ancestral Cadmium* population and the *Grand Ancestor* (|*p_AC_*−*p_GA_*|<0.1) as well as low differentiation between the five replicate *Cad* populations and the initial allele frequency (|*p_Cad_*−*p_ini_*|<0.1, where *p_ini_* = (*p_AC_*+*p_AS_*)/2). Similarly, ∼6% (7498/123291) could be classified as possibly neutral in salt. By this *ad hoc* categorization, only ∼11% of significantly differentiated sites show a pattern consistent with CN.

The discussion above has focused on scenarios (EAS and CN) where selection in heterogeneous environments is intermediate to that of the two homogeneous environments. However, environmental heterogeneity may select for properties not favoured in either constant environment. We see some evidence suggestive of unique sites being favoured by heterogeneity. Among the several thousands of significantly differentiated sites between any pair of heterogeneous and homogeneous treatments (Table 1), about 20% to 35% of such sites are different from the sites differentiated between *Cad* and *Salt* treatments. This means that the divergence of a heterogeneous treatment from a homogeneous treatment is not a simple subset of the divergence between alternative homogenous treatments, suggesting that some sites respond specifically to heterogeneity. Heterogeneity may favor a generalist or plastic genotype (rather than a mix of specialists) and this is thought to be more likely with temporal than spatial heterogeneity [42]. Relative to the *Spatial* treatment, the *Temp* treatment has more significantly differentiated sites from the constant treatments (23745 vs. 9920) and these sites are less likely to be differentiated between the two constant treatments (9147/23745 = 39% of sites differentiated between *Temp* and the constant treatments are not differentiated between the two constant treatments; 2278/9920 = 23% for *Spatial*; χ^2^ = 754.7179, df = 1, p-value<2.2e-16).

Understanding the mechanisms maintaining genetic variation is a classic pursuit of evolutionary biology. Environmental heterogeneity has long been postulated to sustain elevated levels of variation through antagonistic pleiotropy between environments, which can result in balancing selection especially if heterogeneity is spatial rather than temporal. However, rather than antagonism, different loci may be selected in each environment (i.e., conditional neutrality). Further, heterogeneity may select for alleles different from those favored in either single habitat (e.g., generalist genotype rather than a mixture of specialists). These three distinct, but not mutually exclusive, possibilities create uncertainty in how environmental heterogeneity will affect genetic variation. Although there is indirect evidence of each of these genetic possibilities in our data, the major patterns are most consistent with environmental antagonism. However, even consideration of EAS alone does not lead to a single prediction because the effect of environmental heterogeneity on neutral sites depends on their linkage to selected sites.

Although the patterns of diversity among treatments are most consistent with the prediction from EAS, it is likely that some populations are not at equilibrium and these patterns could change over time. For example, we expect CN sites specific to each environment would lose variation in heterogeneous treatments but the rate of loss would be slower than in the appropriate homogenous environment where they experience constant selection. Thus, the relative contributions of CN and EAS sites to diversity will change over time and at different rates across treatments. Given that the current patterns appear to be dominated by EAS and that the contribution of CN is expected to decline over time, it seems unlikely that the patterns would change dramatically through this effect. Nonetheless, it would be ideal to re-sequence these populations at several time points in the future. From the changes in allele frequencies during a time series, we could gain a better sense the effects of environmental heterogeneity on the genetic variation and how these change over time.

This experiment serves as a case study of the effects of environmental heterogeneity on genome-wide variation in a simplified setting, demonstrating distinct differences between environmental heterogeneity in space versus time and between sites likely to be closely linked or not to sites under differential ecological selection (Figure 5). Yet this experiment is only a single test of environmental heterogeneity at one time point; different environments, different spatial or temporal scales of heterogeneity, or different organisms could yield different patterns because the results will depend on the genetic basis of adaptation and the nature of selection [43]. A recent study in yeast suggests that antagonistic effects may be common [44]. But other field studies in plants found that patterns of conditional neutrality are more common [45], [46]. A recent study in Brassicaceae quantified the proportion of conditional neutral and EAS QTLs across genome and found that conditional neutrality is more common than EAS (8% vs. 3% of the genome, [47]). The ultimate challenge remains to determine how environmental variation affects patterns of diversity and quantitative genetic variation in nature in different organisms (e.g., [48]). Because the constraints of real systems make it difficult to cleanly test these effects in nature, experimental evolution provides a helpful step towards testing the key principles [49].

## Methods

### History of selection populations

The selective histories of all populations we used are illustrated in Figure 1. A population of *Drosophila melanogaster* was collected in the Similkameen Valley, British Columbia in 2005 and maintained in regular benign conditions at large size (∼2000–4000 adults, by S. Yeaman); we refer to this population as the “*Grand Ancestor*” (*GA*). In July 2007, a subset of flies from the *GA* population was used to initiate 12 replicate populations (each has ∼1000 adults) maintained in a cadmium-enriched environment (by C.C Spencer); In April 2008, the 12 replicate populations were pooled to generate a single populations maintained in cadmium environment (with a population size of ∼2000 adults), we refer to this as the “*Ancestral Cadmium*” (*AC*) population. In August 2008, a subset of flies from the *GA* population was used to initiate a population (with a population size of ∼2000 adults) maintained in a salt-enriched environment (by A. Wang); we call this the “*Ancestral Salt*” (*AS*) population. During the adaptive history, the amount of cadmium and salt in the environments were progressively increased, reaching 75 µg/ml and 29 mg/ml, respectively at the time to start the experimental evolution project. In October 2009, we collected 448 males and 448 virgin females from both the *AC* and *AS* populations and crossed flies from different populations via mass mating. Flies were collected from the next generation and randomly divided them into four selection regimes (by T.A.F. Long): constant salt-enriched environment (*Salt*), constant cadmium enriched-environment (*Cad*), alternatively in a salt environment or cadmium environment generation by generation (*Temp*), and spatially in either salt or cadmium environment for each generation (*Spatial*). For the *Spatial* treatment, we ensured that the same number of adult flies produced by the two environments contributed to the next generation (i.e., this can be considered a “soft” selection regime *sensu* [50]. Each selection regime had five replicate populations with a population size of 448. Further details of the maintenance of these populations are described in Long et al [25].

### Phenotypic measurements

To confirm that the populations under constant selection adapted to their own environment and to test the fitness of populations under fluctuating selection, we measured the egg to adult viability of flies from these populations in both salt and cadmium testing environments (75 µg/ml cadmium or 22 mg/ml salt). Before the assay, flies were reared in regular benign condition for one generation to control the maternal environment. We mated each virgin female with one male for each population and then let mated females lay eggs in vials with salt food or cadmium food for about 18 hours and 11.5 to 12 days later scored the number of adult flies from each vial. There were ∼140 vials per population per environment for measuring survival.

### Population resequencing

At generation 42 we sampled 70 adult female flies from each of the 23 populations (20 treatment populations plus the three source populations, *AS*, *AC*, and *GA*). For each population, we pooled the females to extract genomic DNA for next-gen sequencing. We obtained 100 bp paired-end short reads from Illumina HiSeq 2000. Paired-end reads were aligned to the *D. melanogaster* r. 5.42 genome sequences using bwa v. 0.5.9 [51] with default settings and the “-I” option. The alignments were filtered using a mapping quality and base quality (PHRED quality score) of 20 as cutoff by samtools v. 0.1.16 [52] and popoolation2 [53]. After the filtering, the range of coverage among 23 populations for euchromatic regions is 12.2∼26.7.

### Allele frequency differentiation between treatments

To look for differentiation between salt and cadmium environments, we first screened for sites in euchromatic regions with at least 5-fold coverage in both ancestral populations (*AS* and *AC*) as well as in the five *Salt* and five *Cad* populations. We kept only those sites where the initial diversity was not too low, specifically π*_ini_* = 2p*_ini_* *(1-p*_ini_*)>5%, where p*_ini_* is the frequency of the minor allele pooling across the two ancestral populations (*AS* and *AC*). After applying these screens, there were ∼2*10^6^ sites for testing differentiation; we refer to these sites as the “α-sites”. We considered allele frequency data from the five replicate *Salt* populations and the *Ancestral Salt* population as six replicates for a “salt” treatment and the data from the five replicate *Cad* populations and the *Ancestral Cadmium* population as six replicates for a “cadmium” treatment. The significant differentiated sites between “cadmium” treatment and “salt” treatment were identified by Cochran-Mantel-Haenszel (CMH) test [26] in R (version 2.15.1 R-Development-Core-Team 2012). The CMH test is a paired test and it was sensible to pair *AC* with *AS* but the pairings of the replicate *Cad* and *Salt* populations are arbitrary. Consequently, we repeated the analysis for each SNP five times, each time pairing *AC* with *AS* but using different (and unique) pairings for the replicate *Cad* and *Salt* populations (1^st^ pairing: *Cad*1 vs *Salt*1, *Cad*2 vs *Salt*2, *Cad*3 vs *Salt*3, *Cad*4 vs *Salt*4, *Cad*5 vs *Salt*5; 2^nd^ pairing: *Cad*1 vs *Salt*2, *Cad*2 vs *Salt3*, *Cad*3 vs *Salt*4, *Cad*4 vs *Salt*5, *Cad*5 vs *Salt*1; …). For the pairwise genetic differentiation between treatment pairs, we did similar tests for different and unique five pairings between five replicate populations from one treatment and those from the another treatment, without the *AC* and the *AS*.

The q-value for each site was calculated from the geometric mean p-value of the five different tests and converted to q-value via the “BY” method in the p.adjust package in R [54]. To show the differentiation across genome, we used a 5 kb sliding-window with a step size of 5 kb to calculate the average -log(q-value) and allele frequency differentiation (based on the environment-specific ancestor and the five replicate populations) for sites within windows (Figure 3A, 3B, Supplementary Information S1A and Figure S1). All the figures except Figure 1 were generated using the R library gglot2 [55]. A SNP was only considered differentiated (i.e., a β-site) if it passed a significance cutoff in all five tests (false discovery rate = 0.001% for q-value from each test). To evaluate the potential for false positives including sampling error, allele frequency estimation error or genetic drift, we randomly assigned half of our cadmium populations as well as half of our salt populations to pseudo-treatment “A” and the remaining cadmium and salt populations to pseudo-treatment “B”. We then performed the same analysis looking for differentiation between “A” and “B”. We repeated this process for 15 randomly chosen combinations.

We examined the enrichment of genic SNPs relative to intergenic SNPs for different -log (q-value) bins [56], [57]. Using all the “α-sites”, we calculated the ratio of number of sites located in genic and intergenic region for each -log(q-value) bin. In order to compare the enrichment across different functional categories, we standardized the ratios relative to the mean ratio across the 11 bins. We performed similar enrichment analyses for other functional categories: coding sequences/intron sites, 0-fold sites/4-fold sites in coding region.

Genes overlapping with at least one significant SNP (β-sites) and their Gene Ontology annotations were identified using the FlyBase annotation (release 5.43) [58]. Gene Ontology enrichment tests were performed using Gowinda [59] with β-sites as significant sites and α-sites as background sites and these parameters: simulations: 100000; min-significance: 1; gene-definition: gene; threads: 8. We repeated the enrichment tests for each of the two major autosomes chromosomes separately. The levels of linkage disequilibrium (LD) within two pair-ends (∼250 bp) were estimated by the program LDx [60].

The frequency of each known inversion was estimated from the average frequency of all inversion-specific SNP markers [31], weighted by the coverage on each marker. The high and low recombination rate regions were divided based on the estimations in [61]. The high regions were defined as having a recombination rate greater than 2 cM/Mb.

### Molecular diversity

Genome-wide diversity (measured by Tajima's π) was calculated via the program Popoolation [35], [36]. After trimming and mapping the reads (quality threshold 20, min-length 60), we used 10 kb non-overlapping sliding windows across genome for each population. To be included in the analysis, we required that at least 60% of sites in a window have at least 4-fold coverage (parameter: window-size 10000 –step-size 10000 –min-count 2 –min-coverage 4 –max-coverage 400 –min-qual 20 –pool-size 140). There were 11265 common windows that passed the cutoff for all 23 populations, covering about 95% of the euchromatic region of the genome.

To calculate allele frequency differentiation and diversity, we screened sites that had at least 15-fold coverage in both ancestral populations (*AC* and *AS*) and had π*_ini_*>5% (estimated from the allele frequency in the *AC* and *AS*), based on the synchronized file generated by Popoolation2 [53]. Also, we screened for the sites that had at least 10-fold coverage in all 20 treatment populations. Further, we calculated the local median coverage among the two ancestral population and 20 treatment populations for all sites in euchromatic region and then the global median among these local medians. We excluded the sites whose local median coverage is higher than two fold of the global median coverage. In total, we had 769,924 SNPs (χ-sites) for the diversity analysis. The differentiation between *AC* and *AS* population (*d*) for each χ-site was calculated via the allele frequencies in *AC* and *AS* populations: |*p_AC_*−*p_AS_*|. The π for each χ-site in was calculated as: π = 1−((A*A)+(G*G)+(C*C)+(T*T)+(D*D)), where A, G, C, T, D are the frequencies of different bases at that site, where D represents a deletion. To control for initial diversity, we screened for high initial diversity (π*_ini_*>0.4) from the χ-sites and had 86,629 sites from which to recalculate the π in each *d* category for each treatment.

### *F_ST_* for replicates within each treatment

To assess the divergence among replicates within each treatment, we measured *F_ST_* among the five replicate populations within each treatment. From the χ-sites, we screened for those with high initial diversity (π*_ini_*>0.4). The average expected heterozygosity (*H_S_*) and total heterozygosity (*H_T_*) for each site within each treatment was calculated as follows:
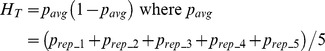


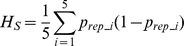
Following Nei 1977 from [62], the mean *F_ST_* over all sites as
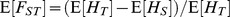
First, we calculated the E[*F_ST_*] for sites located in the two major autosomes and X chromosome for each of the four treatments using putatively neutral sites (i.e., only sites that were weakly differentiated between the ancestral populations (*d* = |*p_AC_*−*p_AS_*|<0.3)). We performed a paired t-test to test the *F_ST_* difference between autosomes and X, pairing the data from the same treatment.

To compare the *F_ST_* among treatments statistically, we selected five regions of the genome that approximately recombinationally independent. The sequence location for these regions are: 2L from 1 to 7,307,159, 2R from 10,368,692 to the end, 3L from 1 to 7,753,553, 3R from 17,055,561 to the end and X chromosome. The two regions on the second (third) chromosome are separated from each other by 50.1 (47.7) cM. Because differences in coverage among treatments could result in artificial differences in *F_ST_* [63], we used an equivalent level of coverage to measure *F_ST_* for each treatment. To do so, we performed the following procedure for each site. We first ranked by coverage the five replicate populations within treatments. For each rank *i* (*i*∈[1], [5]), we found the minimum coverage across treatments, *n_i_*. We sampled without replacement *n_i_* alleles from the *i^th^* ranked population for each treatment. Based on these resampled allele frequencies, we calculated the average *F_ST_* based on the equation above for each treatment. We repeated the whole resampling and calculation 10 times and used the mean *F_ST_* among the 10 results for statistical analysis. We used ANOVA and TukeyHSD test to compare the difference in *F_ST_* among treatments.

## Supporting Information

Figure S1 Genetic differentiation (-log(q-value)) across chromosome arms. Sliding windows (5 kb non-overlapping) showing the average -log(q-value) per window across the three major chromosomes (see Method). Higher -log(q-value) indicates higher differentiation between populations in salt and cadmium environments.
(TIFF)

Figure S2 The ratio of number of 0-fold to 4-fold degenerate sites in different q-value bins. The higher value of the q-value bins, the higher differentiation between *Salt* and *Cad* populations. Tn order to compare the manitude for different functional catergories, the ratios are standardized around 1 by dividing the mean ratio among the 11 bins.
(TIFF)

Figure S3 The fraction of β-sites among α-sites within windows around focal β-sites. The y-axis is the average fraction of β-sites among α-sites in windows around 5000 randomly selected β-sites. The x-axis is the log (in base 10) of the length of the window. For some small windows, there are no α-sites so the fraction cannot be calculated. For windows sizes of 50, 100, 500, 1000, 5000, 10000 and 50000 bps, the number of useable focal sites were 3308, 4111, 4971, 4992, 5000, 5000, and 5000, respectively. The true data are shown in blue and the permuted data (null distribution under no clustering) are shown in red. The error bars are 95% confidence intervals.
(TIFF)

Figure S4 The mean *F_ST_* between salt and cadmium environments as function of distance from focal sites. *F_ST_* values are calculated by sliding windows moving from the focal sites (∼1000 focal sites, sites identified as being q-value significantly divergent between environments (A)). For each focal site, we used non-overlapping 50 base pair windows moving away from the position of the site to 2000 bp for both directions. We calculated the mean *F_ST_* for all variants within each window weighted by each of their total variance among all the populations. The *F_ST_* values for windows with the same distance were averaged among all the focal sites. Focal sites were either randomly chosen (differentiated) β-sites (A) or randomly chosen non-significant sites (B). The number of windows for different distances used in calculating the average *F_ST_* is in the range of 700–960; some windows have low molecular variance and are excluded. For the first window, starting at distance 0, we excluded the focal site from the calculation.
(TIFF)

Figure S5 The linkage disequilibrium (measured as *r* ^2^) between two SNPs within pair-end reads. The 1^st^ to 4^th^ rows are replicate populations of *Salt*, *Cad*, *Temp* and *Spatial* treatments, respectively. The last three plots are *Grand Ancestor*, *Ancestral Salt* and *Ancestral Cad* population. Each point is the average *r* ^2^ among pairs of SNPs within each 5 bp window for different distance. The blue dots are results for all SNP pairs that pass the LDx screening. The open red circles are results for the SNP pairs within which at least one significantly differentiated site (β-site) exists. These two results are not obviously different from each other, except in the *Ancestral Salt* and *Ancestral Cad* populations where the *r* ^2^ seems to be lower for pairs involving significant sites. Overall, the *Grand Ancestor* (the first column in the last row) tends to have lowest *r* ^2^ among all populations, which is expected as it is the source population for the others.
(TIFF)

Figure S6 Mean π within treatments as function of differentiation (*d*) between *AC* and *AS* for χ-site outside inversions. The average π across different levels of ancestral differentiation for (A) all χ-site SNPs or (B) only those χ-site SNPs that have high initial diversity (π_ini_>0.4). The *x*-axis is the allele frequency difference between ancestral populations, *d* = |*p_AC_*−*p_AS_*|. Error bars represent the standard error among the five replicates for each treatment.
(TIFF)

Figure S7 Simulation results for the variance (π) at a neutral site as a function of its effective recombination distance to selected site. The neutral site is linked to 40 selected site for which selection coefficient s = 0.02 (A), s = 0.05 (B) and *s* = 0.07 (C). The random LD among the distanced selected sites was generated by “clusterGeneration” package in R [64]. The haplotype frequencies were calculated from the allele frequency and the LD among loci [65] (see Supplementary information S5 for details). The x-axis “log(*r*)” stands for the log (in base 10) of the recombination distance (*r*) for the neutral sites to other selected loci, calculated from the harmonic mean physical distance. Each point represents the average of 10,000 simulations per treatment. The error bars are the ±standard error for the replicates. The average variance (π) for the 40 selected loci (not shown in plot) are: (A) π = 0.32 for *Spatial*, 0.28 for *Temp*, 0.076 for *Salt*, 0.086 for *Cad* and 0.29 for the no-selection treatment;(B) π = 0.45 for *Spatial*, 0.31 for *Temp*, 0.0017 for *Salt*, 0.0015 for *Cad* and 0.30 for the no-selection treatment; (C) π = 0.49 for *Spatial*, 0.31 for *Temp*, 0.00045 for *Salt*, 0.00042 for *Cad* and 0.29 for the no-selection treatment.
(TIFF)

Figure S8 Average diversity (π) for high recombination regions (A) and low recombination regions (B) for each population. Based on the estimations in [61], the high and low region were divided using a cutoff of 2 cM/Mb. The average diversity for each region for each population was calculated by Popoolation program [35], [36].
(TIFF)

Figure S9 Average diversity low (*L*) and high (*H*) recombination regions for each population using only those with high initial diversity. The χ-sites with initial diversity (π) >0.4 were divided into low and high recombination categories. The average π for sites in low recombination and high recombination regions was then calculated. There is a significant difference in diversity between low and high regions in *Cad* treatment (paired *t*-test: *t* = 8.1, df = 4, p-value = 0.0013). However, the diversity does not differ between the *H* and *L* regions in the other three treatments.
(TIFF)

Table S1 Significantly enriched functional annotation groups. Enrichment tests were performed using the whole genome, only genes on chromosome 2 and only genes on chromosome 3. Note that differences in the numbers of genes located in different chromosomes results in differences in statistical power for detecting enrichment for each GO category between chromosomes. For simplicity, only a subset of enriched GO terms are shown.
(DOCX)

Table S2 The frequencies of inversions within the ancestral populations and for each treatment. The frequency for each inversion within populations is estimated from the average frequency across all inversion-specific alleles within the inversion, weighted by the coverage for each of these polymorphic sites. There are five inversions exist in our populations. The numbers of sites related to the inversion-specific alleles used in estimations are: In(2L)t: 16; In(2R)Ns: 67; In(3L)P: 73; In(3R)C: 144; In(3R)Mo: 150. The mean and standard error of frequency for each inversion among five replicate populations within treatments are shown.
(DOCX)

Table S3 The allele frequency difference between cadmium and salt environments for significant differentiated sites. The significant differentiated β-sites are divided into inside and outside inversion for each autosome arms, based on the five inversions identified in Table S2. We calculate the average difference in mean allele frequency between cadmium populations (*AC* and five replicate *Cad* populations) and salt populations (*AS* and five replicate *Salt* populations) for different regions in autosomes. The values within brackets show the 2.5% lowest and highest differences among β-sites for each region.
(DOCX)

Table S4 The enrichment of significant sites inside and outside inversion for each chromosome arm. Considering all the significant site (β-sites) and total SNPs (α-sites) over two major autosomes, the proportions of β-sites to α-sites are similar inside and outside the potential inversion regions, with higher enrichment of significant sites for outside the inversion (0.054 for inside vs 0.072 for outside).
(DOCX)

Table S5 The average *r* ^2^ inside and outside inversion for each chromosome arm. Average *r* ^2^ was calculated first average *r* ^2^ within 150 bp base pair for each 5 bp window (as describe in Supplementary Information S2C) for different chromosome arms, separating the regions inside and outside inversion. Then we calculated the average *r* ^2^ value among the *AS*, *AC* and five replicate *Salt*, five replicate *Cad* (the 12 populations used to identify β-sites). The average *r* ^2^ among the four chromosome arms are similar inside and outside inversions (0.454 vs 0.451). For separate chromosome arms, the differences in *r* ^2^ inside and outside inversion correlate with the differences in proportion of β-sites to α-sites.
(DOCX)

Table S6 The enrichment of significant sites (β-sites) in low recombination and high recombination. We divided the genome into low and high recombination rate regions. Based on the estimations in [61], the high region was defined as having a recombination rate greater than 2 cM/Mb. We calculated the number and proportion of β-sites and α-sites in each of these regions. There is a higher proportion of significant sites to α-sites in low recombination regions than high recombination regions (7.6% vs 5.4%, the difference is caused by autosome 2).
(DOCX)

Table S7 Numbers of genes that overlap with at least one significantly differentiated sites between different pairs of treatments. The significantly differentiated sites are identified based on the genetic differentiation between treatment pairs, using the five replicate populations from one treatment and those from the other treatment. The Gene Ontology annotations were identified using the FlyBase annotation (release 5.43) [58].
(DOCX)

Table S8 The difference in correlation between significant site and control sites in treatment pairs (Diff_Cor). Allele frequencies for each site were averaged across replicates of each treatment. The Pearson's product-moment correlation in average allele frequency was calculated between each pair treatments for putatively selected sites as well as for control sites. The difference between the two correlations is Diff_Cor. The final column shows the Diff_Cor value for each treatment with the initial allele frequency, *p_ini_* = (*p_AS_*+*p_AC_*)/2. See Supplementary Information S3 for details.
(DOCX)

Table S9 Average diversity (± SE) for the high and low recombination regions in each treatment using all sites. The standard errors were calculated from the point estimations from the five replicate populations within treatments. These data are also plotted in Figure S8.
(DOCX)

Supplemental Information S1 Evidence of multiple targets of selection underlying differentiation between salt and cadmium environments.
(DOCX)

Supplemental Information S2 Evidence of linkage effects contributing to differentiation between salt and cadmium environments.
(DOCX)

Supplemental Information S3 Calculation of allele frequency correlations between treatments.
(DOCX)

Supplemental Information S4 Diversity patterns based on the χ-sites outside of possible inversion regions.
(DOCX)

Supplemental Information S5 Simulations of experimental populations.
(DOCX)

Supplemental Information S6 Diversity in regions of high and low recombination.
(DOCX)
