# Supplementary figures and images for: Genome-Wide Patterns of Genetic Variation within and among Alternative Selective Regimes
Source: PLoS Genet. 2014 Aug 7;10(8):e1004527. doi: 10.1371/journal.pgen.1004527 (PMC4125100; doi:10.1371/journal.pgen.1004527)

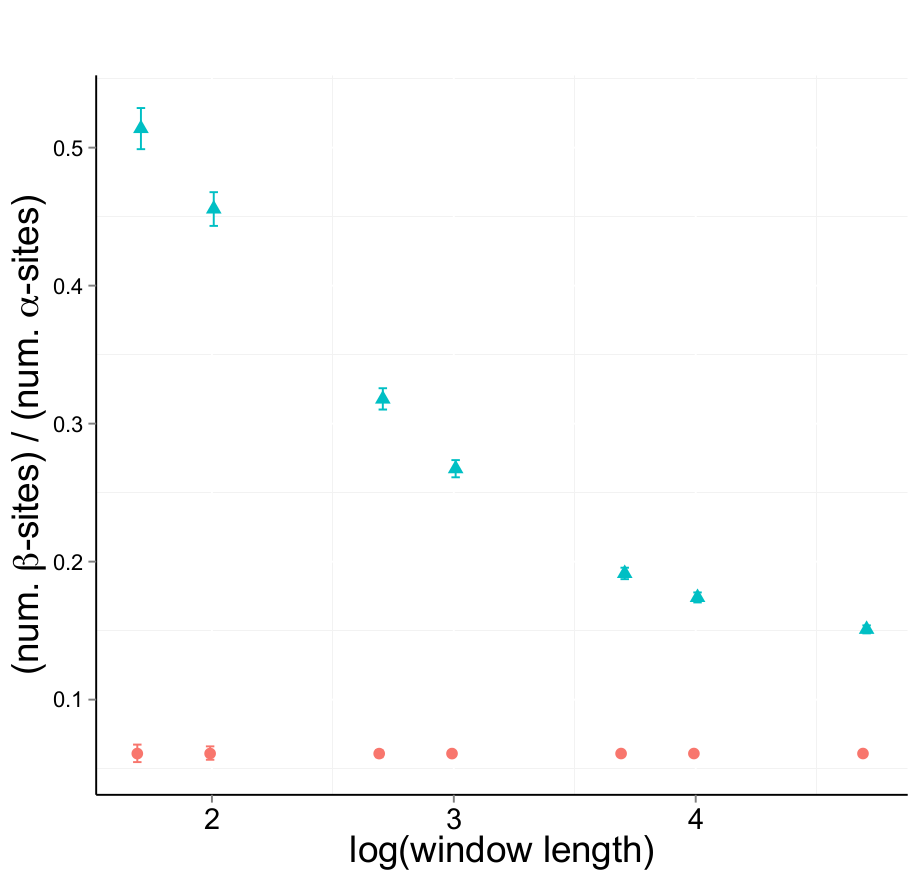

Supplement: Figure S3 — The fraction of β-sites among α-sites within windows around focal β-sites. The y-axis is the average fraction of β-sites among α-sites in windows around 5000 randomly selected β-sites. The x-axis is the log (in base 10) of the length of the window. For some small windows, there are no α-sites so the fraction cannot be calculated. For windows sizes of 50, 100, 500, 1000, 5000, 10000 and 50000 bps, the number of useable focal sites were 3308, 4111, 4971, 4992, 5000, 5000, and 5000, respectively. The true data are shown in blue and the permuted data (null distribution under no clustering) are shown in red. The error bars are 95% confidence intervals. (TIFF) [file pgen.1004527.s003.tiff]

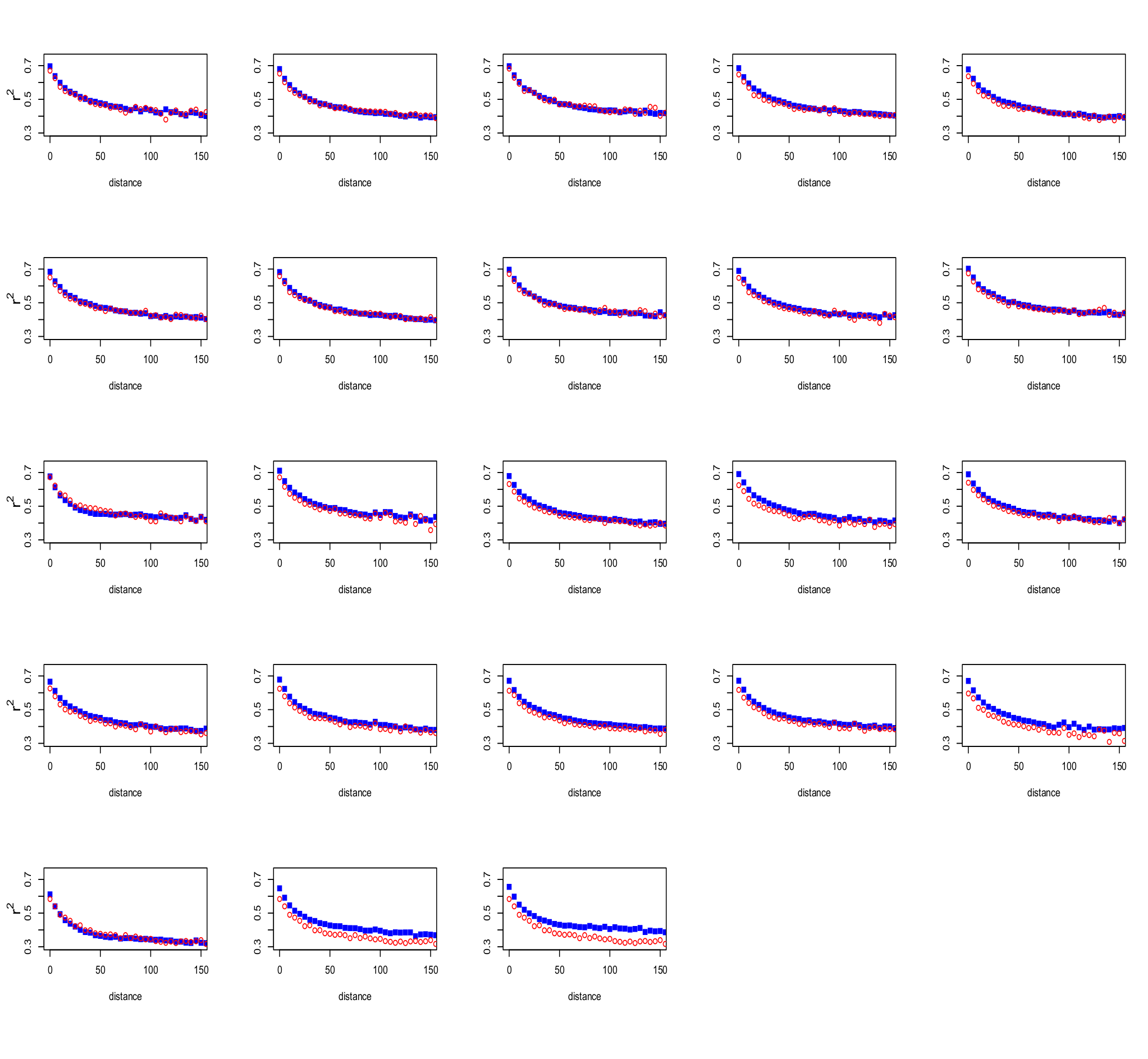

Supplement: Figure S5 — The linkage disequilibrium (measured as r 2) between two SNPs within pair-end reads. The 1st to 4th rows are replicate populations of Salt, Cad, Temp and Spatial treatments, respectively. The last three plots are Grand Ancestor, Ancestral Salt and Ancestral Cad population. Each point is the average r 2 among pairs of SNPs within each 5 bp window for different distance. The blue dots are results for all SNP pairs that pass the LDx screening. The open red circles are results for the SNP pairs within which at least one significantly differentiated site (β-site) exists. These two results are not obviously different from each other, except in the Ancestral Salt and Ancestral Cad populations where the r 2 seems to be lower for pairs involving significant sites. Overall, the Grand Ancestor (the first column in the last row) tends to have lowest r 2 among all populations, which is expected as it is the source population for the others. (TIFF) [file pgen.1004527.s005.tiff]

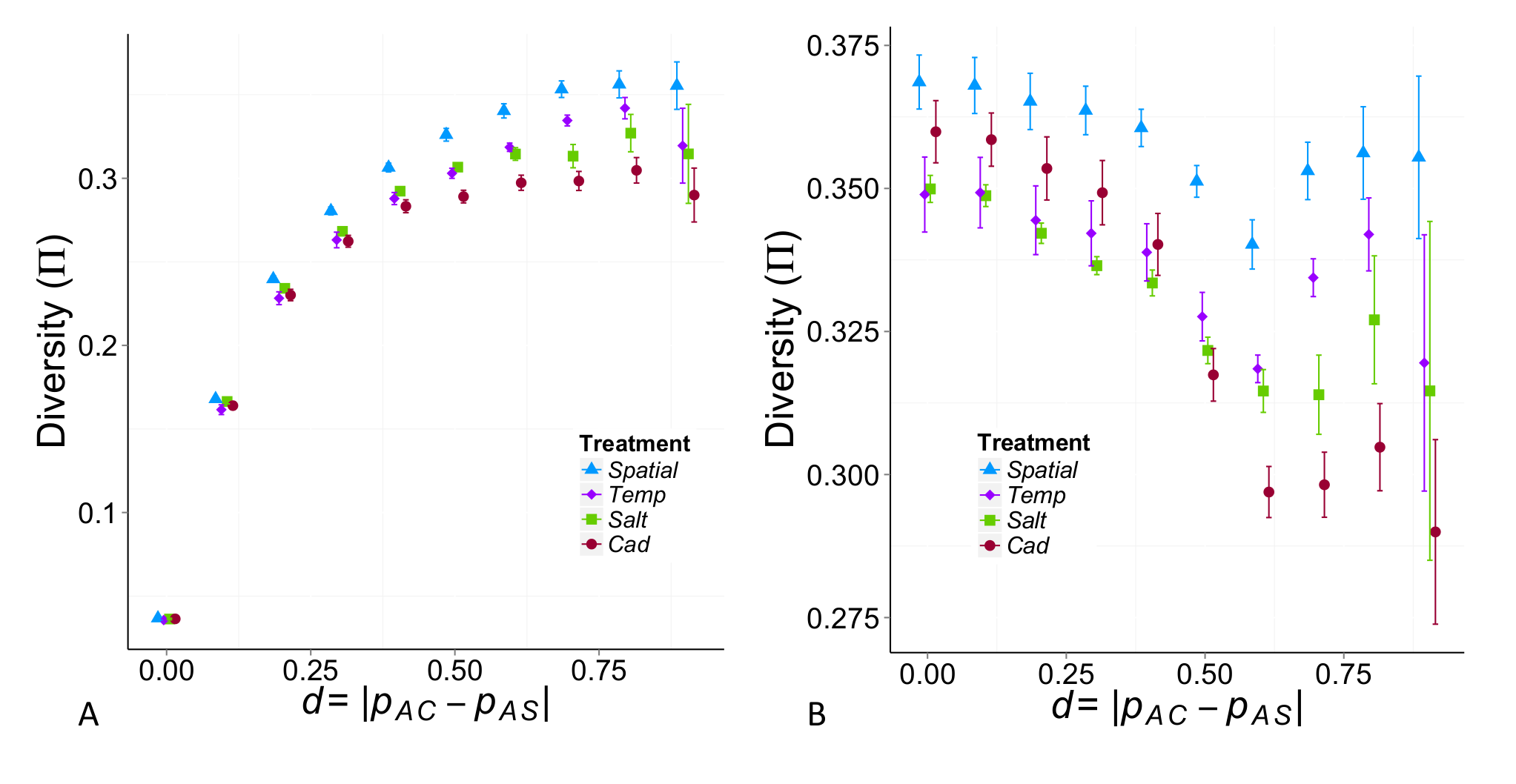

Supplement: Figure S6 — Mean π within treatments as function of differentiation (d) between AC and AS for χ-site outside inversions. The average π across different levels of ancestral differentiation for (A) all χ-site SNPs or (B) only those χ-site SNPs that have high initial diversity (πini>0.4). The x-axis is the allele frequency difference between ancestral populations, d = |pAC−pAS|. Error bars represent the standard error among the five replicates for each treatment. (TIFF) [file pgen.1004527.s006.tiff]

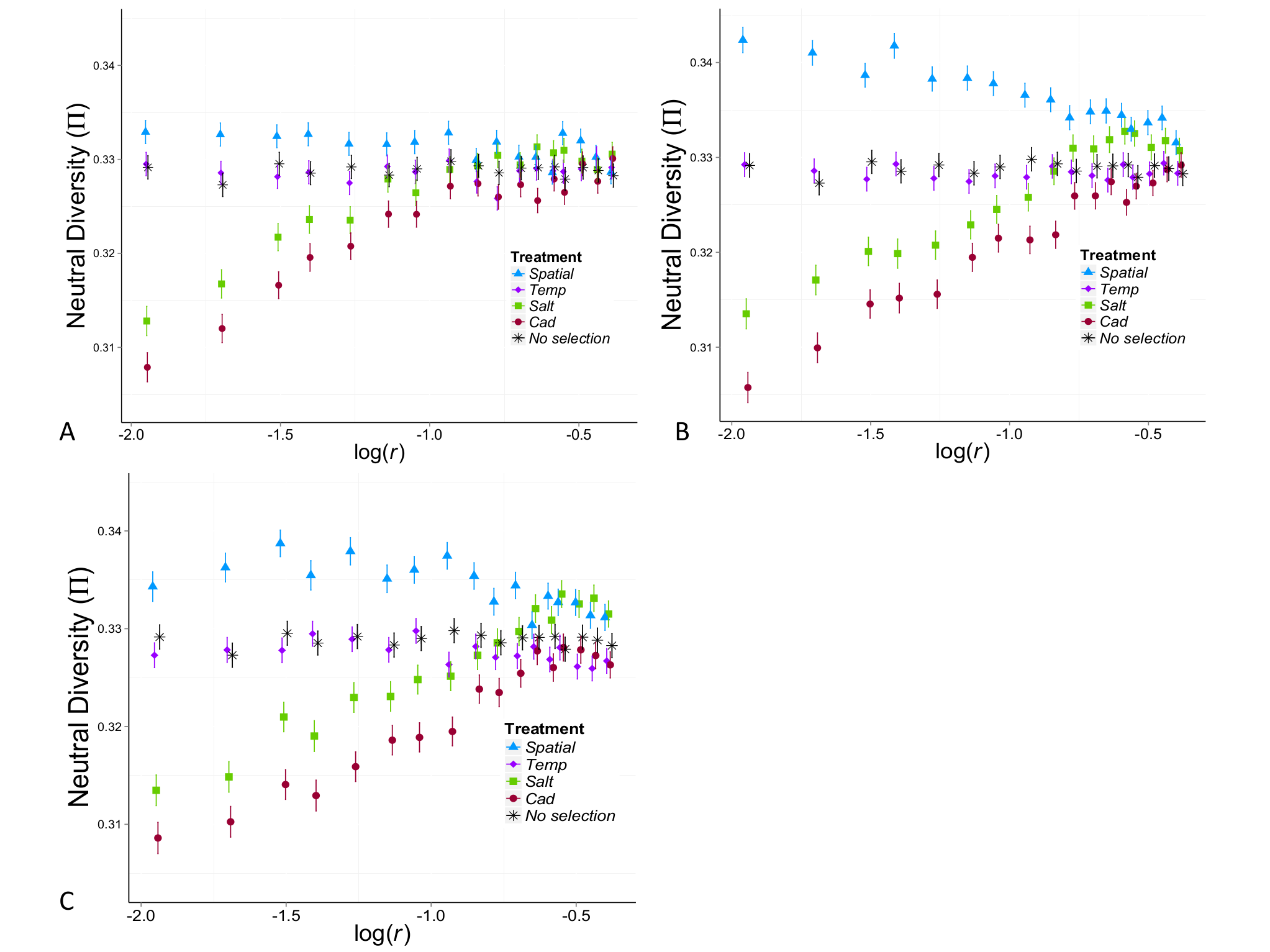

Supplement: Figure S7 — Simulation results for the variance (π) at a neutral site as a function of its effective recombination distance to selected site. The neutral site is linked to 40 selected site for which selection coefficient s = 0.02 (A), s = 0.05 (B) and s = 0.07 (C). The random LD among the distanced selected sites was generated by “clusterGeneration” package in R [64]. The haplotype frequencies were calculated from the allele frequency and the LD among loci [65] (see Supplementary information S5 for details). The x-axis “log(r)” stands for the log (in base 10) of the recombination distance (r) for the neutral sites to other selected loci, calculated from the harmonic mean physical distance. Each point represents the average of 10,000 simulations per treatment. The error bars are the ±standard error for the replicates. The average variance (π) for the 40 selected loci (not shown in plot) are: (A) π = 0.32 for Spatial, 0.28 for Temp, 0.076 for Salt, 0.086 for Cad and 0.29 for the no-selection treatment;(B) π = 0.45 for Spatial, 0.31 for Temp, 0.0017 for Salt, 0.0015 for Cad and 0.30 for the no-selection treatment; (C) π = 0.49 for Spatial, 0.31 for Temp, 0.00045 for Salt, 0.00042 for Cad and 0.29 for the no-selection treatment. (TIFF) [file pgen.1004527.s007.tiff]

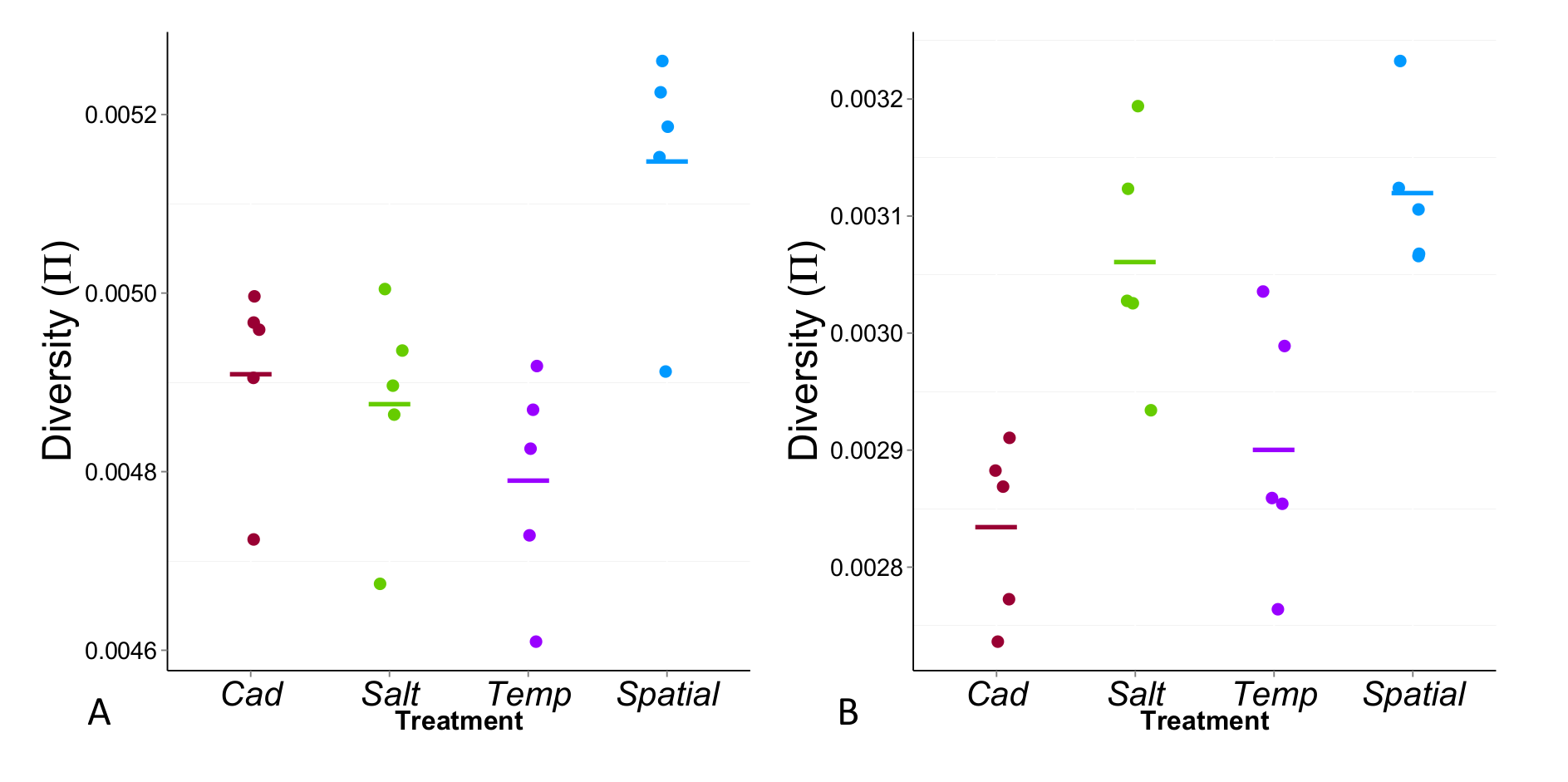

Supplement: Figure S8 — Average diversity (π) for high recombination regions (A) and low recombination regions (B) for each population. Based on the estimations in [61], the high and low region were divided using a cutoff of 2 cM/Mb. The average diversity for each region for each population was calculated by Popoolation program [35], [36]. (TIFF) [file pgen.1004527.s008.tiff]

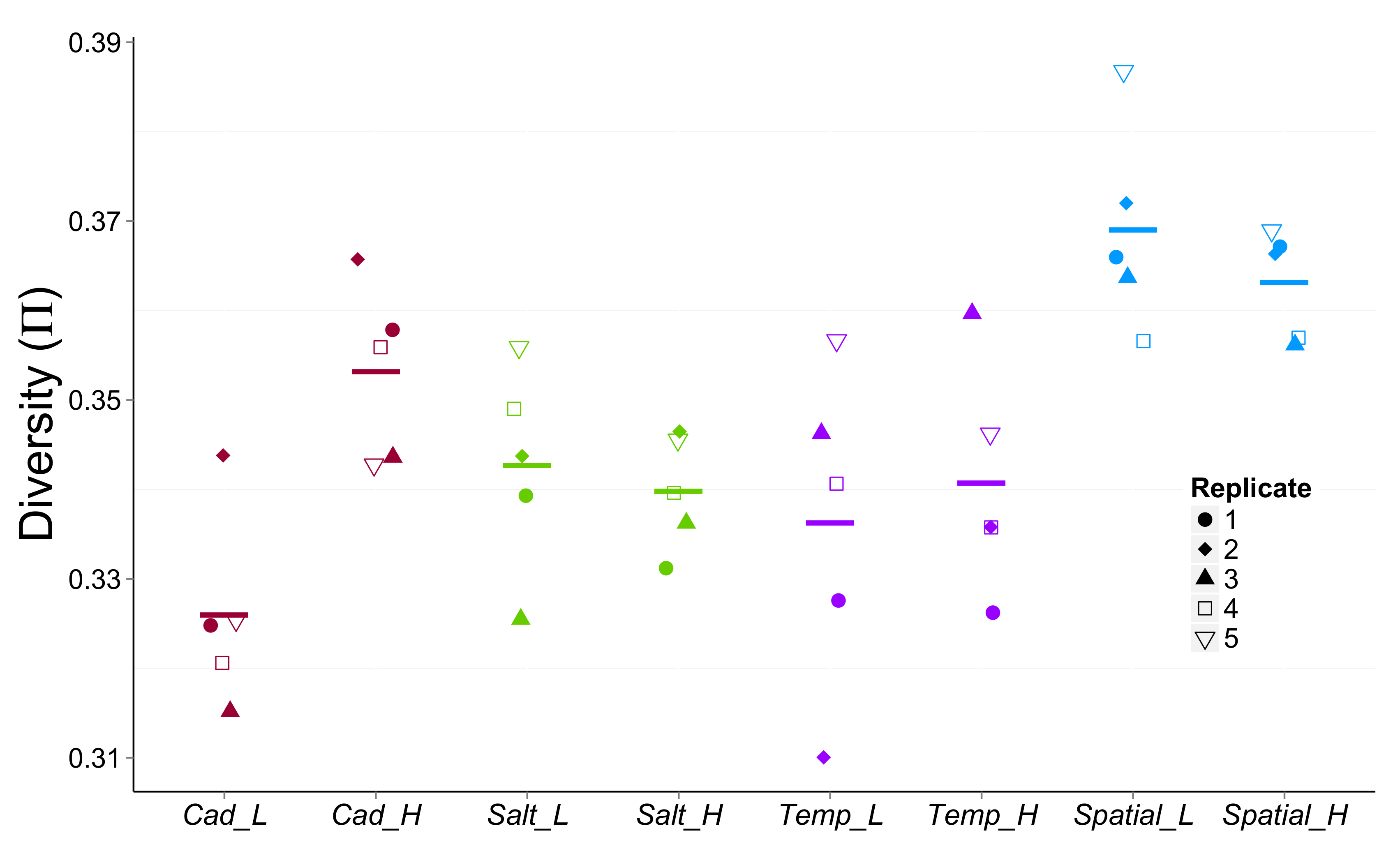

Supplement: Figure S9 — Average diversity low (L) and high (H) recombination regions for each population using only those with high initial diversity. The χ-sites with initial diversity (π) >0.4 were divided into low and high recombination categories. The average π for sites in low recombination and high recombination regions was then calculated. There is a significant difference in diversity between low and high regions in Cad treatment (paired t-test: t = 8.1, df = 4, p-value = 0.0013). However, the diversity does not differ between the H and L regions in the other three treatments. (TIFF) [file pgen.1004527.s009.tiff]
