# Supplementary material for: Genome-Wide Patterns of Genetic Variation within and among Alternative Selective Regimes
Source: PLoS Genet. 2014 Aug 7;10(8):e1004527. doi: 10.1371/journal.pgen.1004527 (PMC4125100; doi:10.1371/journal.pgen.1004527)
Supplement: Table S1 — Significantly enriched functional annotation groups. Enrichment tests were performed using the whole genome, only genes on chromosome 2 and only genes on chromosome 3. Note that differences in the numbers of genes located in different chromosomes results in differences in statistical power for detecting enrichment for each GO category between chromosomes. For simplicity, only a subset of enriched GO terms are shown. (DOCX) [file pgen.1004527.s010.docx]

**Table S1. Significantly enriched functional annotation groups**

| **Whole genome** | **q-value** | **Chromosome2** | **q-value** | **Chromosome3** | **q-value** |
| --- | --- | --- | --- | --- | --- |
| protein metabolic process | 0.0008 | metallopeptidase activity | 0.0045 | proteolysis | 0.00125 |
| cellular macromolecule biosynthetic process | 0.0008 | proteolysis | 0.0045 | response to organic substance | 0.00126 |
| peptidase activity | 0.0008 | protein metabolic process | 0.0045 | peptidase activity | 0.00125 |
| macromolecule metabolic process | 0.0008 | regulation of steroid hormone secretion | 0.0057 | peptidase activity, acting on L-amino acid peptides | 0.00125 |
| endopeptidase activity | 0.0008 | endomitotic cell cycle | 0.0086 | translation | 0.00350 |
| regulation of steroid hormone secretion | 0.0008 | detection of pheromone | 0.0100 | cellular macromolecule biosynthetic process | 0.00350 |
| translation | 0.0008 | peptidase activity, acting on L-amino acid peptides | 0.0131 | macromolecule biosynthetic process | 0.00860 |
| proteolysis | 0.0008 | metalloendopeptidase activity | 0.0170 | endopeptidase activity | 0.00980 |
| detection of pheromone | 0.0008 | macromolecule metabolic process | 0.0238 | protein metabolic process | 0.01280 |
| macromolecule biosynthetic process | 0.0008 | peptidase activity | 0.0373 | macromolecule metabolic process | 0.01580 |
| peptidase activity, acting on L-amino acid peptides | 0.0008 | endopeptidase activity | 0.0370 |  |  |
