# Supplementary material for: Genome-Wide Patterns of Genetic Variation within and among Alternative Selective Regimes
Source: PLoS Genet. 2014 Aug 7;10(8):e1004527. doi: 10.1371/journal.pgen.1004527 (PMC4125100; doi:10.1371/journal.pgen.1004527)
Supplement: Table S2 — The frequencies of inversions within the ancestral populations and for each treatment. The frequency for each inversion within populations is estimated from the average frequency across all inversion-specific alleles within the inversion, weighted by the coverage for each of these polymorphic sites. There are five inversions exist in our populations. The numbers of sites related to the inversion-specific alleles used in estimations are: In(2L)t: 16; In(2R)Ns: 67; In(3L)P: 73; In(3R)C: 144; In(3R)Mo: 150. The mean and standard error of frequency for each inversion among five replicate populations within treatments are shown. (DOCX) [file pgen.1004527.s011.docx]

| frequency | *GA* | *AS* | *AC* | *Cad* | *Salt* | *Temp* | *Spatial* |
| --- | --- | --- | --- | --- | --- | --- | --- |
| In(2L)t | 0.1480 | 0.0058 | 0.0000 | 0.048±  0.0122 | 0.001±  0.0007 | 0.008±  0.0072 | 0.069±  0.0262 |
| In(2R)Ns | 0.0020 | 0.0000 | 0.0018 | 0.001±  0.0003 | 0.000±  0.0003 | 0.001±  0.0003 | 0.000±  0.0003 |
| In(3L)P | 0.0026 | 0.0006 | 0.0022 | 0.002±  0.0008 | 0.001±  0.0005 | 0.002±  0.0004 | 0.001±  0.0002 |
| In(3R)C | 0.0049 | 0.0025 | 0.0105 | 0.014±  0.0029 | 0.006±  0.0013 | 0.007±  0.0007 | 0.008±  0.0012 |
| In(3R)Mo | 0.0048 | 0.0444 | 0.0076 | 0.006±  0.0014 | 0.044±  0.0329 | 0.006±  0.0018 | 0.004±  0.0004 |
