# Supplementary material for: Genome-Wide Patterns of Genetic Variation within and among Alternative Selective Regimes
Source: PLoS Genet. 2014 Aug 7;10(8):e1004527. doi: 10.1371/journal.pgen.1004527 (PMC4125100; doi:10.1371/journal.pgen.1004527)
Supplement: Table S3 — The allele frequency difference between cadmium and salt environments for significant differentiated sites. The significant differentiated β-sites are divided into inside and outside inversion for each autosome arms, based on the five inversions identified in Table S2. We calculate the average difference in mean allele frequency between cadmium populations (AC and five replicate Cad populations) and salt populations (AS and five replicate Salt populations) for different regions in autosomes. The values within brackets show the 2.5% lowest and highest differences among β-sites for each region. (DOCX) [file pgen.1004527.s012.docx]

| Allele frequency difference | β-sites inside inversion | β-sites outside inversion |
| --- | --- | --- |
| 2L | 0.431 (0.278, 0.658) | 0.473 (0.292, 0.688) |
| 2R | 0.444 (0.284, 0.626) | 0.420 (0.252, 0.650) |
| 3L | 0.437 (0.260, 0.673) | 0.408 (0.260, 0.582) |
| 3R | 0.423 (0.256, 0.634) | 0.411 (0.230, 0.614) |
| All β-sites sites | 0.438 (0.261, 0.667) | |

**Table S3. The allele frequency difference between cadmium and salt environments for significant differentiated sites**.
