# Supplementary material for: Genome-Wide Patterns of Genetic Variation within and among Alternative Selective Regimes
Source: PLoS Genet. 2014 Aug 7;10(8):e1004527. doi: 10.1371/journal.pgen.1004527 (PMC4125100; doi:10.1371/journal.pgen.1004527)
Supplement: Table S4 — The enrichment of significant sites inside and outside inversion for each chromosome arm. Considering all the significant site (β-sites) and total SNPs (α-sites) over two major autosomes, the proportions of β-sites to α-sites are similar inside and outside the potential inversion regions, with higher enrichment of significant sites for outside the inversion (0.054 for inside vs 0.072 for outside). (DOCX) [file pgen.1004527.s013.docx]

| Chr. arm | total α-sites | α-sites inside inversion | α-sites outside inversion | Proportion significant site (β-sites) to α-sites inside inversion | Proportion significant site (β-sites) to α-sites outside inversion | total significant site (β-sites) to all α-sites |
| --- | --- | --- | --- | --- | --- | --- |
| 2L | 449142 | 253168 | 195974 | 0.027 | 0.138 | 0.075 |
| 2R | 373440 | 102817 | 270623 | 0.053 | 0.068 | 0.064 |
| 3L | 465714 | 310306 | 155408 | 0.079 | 0.046 | 0.068 |
| 3R | 447387 | 227064 | 220323 | 0.058 | 0.034 | 0.046 |
| auto. sum | 1735683 | 893355 | 842328 | 0.054 | 0.072 | 0.063 |
| X | 283892 |  |  |  |  | 0.046 |
| total | 2019575 |  |  |  |  | 0.061 |

**Table S4. The enrichment of significant sites inside and outside inversion for each chromosome arm.**
