# Supplementary material for: Genome-Wide Patterns of Genetic Variation within and among Alternative Selective Regimes
Source: PLoS Genet. 2014 Aug 7;10(8):e1004527. doi: 10.1371/journal.pgen.1004527 (PMC4125100; doi:10.1371/journal.pgen.1004527)
Supplement: Table S5 — The average r 2 inside and outside inversion for each chromosome arm. Average r 2 was calculated first average r 2 within 150 bp base pair for each 5 bp window (as describe in Supplementary Information S2C) for different chromosome arms, separating the regions inside and outside inversion. Then we calculated the average r 2 value among the AS, AC and five replicate Salt, five replicate Cad (the 12 populations used to identify β-sites). The average r 2 among the four chromosome arms are similar inside and outside inversions (0.454 vs 0.451). For separate chromosome arms, the differences in r 2 inside and outside inversion correlate with the differences in proportion of β-sites to α-sites. (DOCX) [file pgen.1004527.s014.docx]

| **Chromosome are** | **Average *r*^2^ inside inversion** | **Average *r*^2^ outside inversion** |
| --- | --- | --- |
| 2L | 0.435 | 0.465 |
| 2R | 0.452 | 0.452 |
| 3L | 0.452 | 0.425 |
| 3R | 0.475 | 0.460 |
| **Avg auto. arms** | **0.454** | **0.451** |

**Table S5. The average *r*^2^ inside and outside inversion for each chromosome arm**
