# Supplementary material for: Genome-Wide Patterns of Genetic Variation within and among Alternative Selective Regimes
Source: PLoS Genet. 2014 Aug 7;10(8):e1004527. doi: 10.1371/journal.pgen.1004527 (PMC4125100; doi:10.1371/journal.pgen.1004527)
Supplement: Table S6 — The enrichment of significant sites (β-sites) in low recombination and high recombination. We divided the genome into low and high recombination rate regions. Based on the estimations in [61], the high region was defined as having a recombination rate greater than 2 cM/Mb. We calculated the number and proportion of β-sites and α-sites in each of these regions. There is a higher proportion of significant sites to α-sites in low recombination regions than high recombination regions (7.6% vs 5.4%, the difference is caused by autosome 2). (DOCX) [file pgen.1004527.s015.docx]

**Table S6**

| Chr. arm | total α-sites | α-sites low rec | α-sites high rec | Proportion β-sites to α-sites low rec | Proportion β-sites to α-sites high rec | total β-sites to all α-sites |
| --- | --- | --- | --- | --- | --- | --- |
| 2L | 449142 | 121765 | 327377 | 0.185 | 0.035 | 0.075 |
| 2R | 373440 | 82918 | 290522 | 0.095 | 0.055 | 0.064 |
| 3L | 465714 | 133299 | 332415 | 0.045 | 0.078 | 0.068 |
| 3R | 447387 | 143218 | 304169 | 0.033 | 0.052 | 0.046 |
| auto. sum | 1735683 | 481200 | 1254483 | 0.085 | 0.055 | 0.063 |
| X | 283892 | 131999 | 151893 | 0.044 | 0.049 | 0.046 |
| total | 2019575 | 613199 | 1406376 | 0.076 | 0.054 | 0.061 |

**Table S6. The enrichment of significant sites (β-sites) in low recombination and high recombination.**
