# Supplementary material for: Genome-Wide Patterns of Genetic Variation within and among Alternative Selective Regimes
Source: PLoS Genet. 2014 Aug 7;10(8):e1004527. doi: 10.1371/journal.pgen.1004527 (PMC4125100; doi:10.1371/journal.pgen.1004527)
Supplement: Table S7 — Numbers of genes that overlap with at least one significantly differentiated sites between different pairs of treatments. The significantly differentiated sites are identified based on the genetic differentiation between treatment pairs, using the five replicate populations from one treatment and those from the other treatment. The Gene Ontology annotations were identified using the FlyBase annotation (release 5.43) [58]. (DOCX) [file pgen.1004527.s016.docx]

**Table S7**

| **Treatment** | *Cad* | *Temp* | *Spatial* |
| --- | --- | --- | --- |
| *Salt* | 5506 | 1471 | 1305 |
| *Cad* |  | 1779 | 802 |
| *Temp* |  |  | 180 |

**Table S7. Numbers of genes that overlap with at least one significantly differentiated sites between different pairs of treatments**.
