# Supplementary material for: Genome-Wide Patterns of Genetic Variation within and among Alternative Selective Regimes
Source: PLoS Genet. 2014 Aug 7;10(8):e1004527. doi: 10.1371/journal.pgen.1004527 (PMC4125100; doi:10.1371/journal.pgen.1004527)
Supplement: Table S8 — The difference in correlation between significant site and control sites in treatment pairs (Diff_Cor). Allele frequencies for each site were averaged across replicates of each treatment. The Pearson's product-moment correlation in average allele frequency was calculated between each pair treatments for putatively selected sites as well as for control sites. The difference between the two correlations is Diff_Cor. The final column shows the Diff_Cor value for each treatment with the initial allele frequency, pini = (pAS+pAC)/2. See Supplementary Information S3 for details. (DOCX) [file pgen.1004527.s017.docx]

**Table S8**

| \|  \| *Cad* \| *Temp* \| *Spatial* \| Initial allele frequency \| \| --- \| --- \| --- \| --- \| --- \| \| *Salt* \| -0.376 \| -0.168 \| -0.154 \| 0.031 \| \| 95% CI \| (-0.438, -0.316) \| (-0.12, -0.216) \| (-0.2, -0.1) \| (-0.03,0.094) \| \| *Cad* \| # \| 0.0134 \| 0.036 \| 0.158 \| \| 95% CI \|  \| (-0.026,0.051) \| (-0.0006,0.07) \| (0.102,0.215) \| \| *Temp* \|  \| # \| 0.08 \| 0.183 \| \| 95% CI \|  \|  \| (0.047,0.112) \| (0.125,0.239) \| \| *Spatial* \|  \|  \| # \| 0.148 \| \| 95% CI \|  \|  \|  \| (0.095,0.199) \| |
| --- | --- | --- | --- | --- | --- | --- | --- | --- | --- | --- | --- | --- | --- | --- | --- | --- | --- | --- | --- | --- | --- | --- | --- | --- | --- | --- | --- | --- | --- | --- | --- | --- | --- | --- | --- | --- | --- | --- | --- | --- | --- | --- | --- | --- | --- |

**Table S8. The difference in correlation between significant site and control sites in treatment pairs (Diff_Cor).**
