# Supplementary material for: Genome-Wide Patterns of Genetic Variation within and among Alternative Selective Regimes
Source: PLoS Genet. 2014 Aug 7;10(8):e1004527. doi: 10.1371/journal.pgen.1004527 (PMC4125100; doi:10.1371/journal.pgen.1004527)
Supplement: Table S9 — Average diversity (± SE) for the high and low recombination regions in each treatment using all sites. The standard errors were calculated from the point estimations from the five replicate populations within treatments. These data are also plotted in Figure S8. (DOCX) [file pgen.1004527.s018.docx]

**Table S9**

| Treatment | π in High Recombination Rate Regions | π in Low Recombination Rate Regions |
| --- | --- | --- |
| *Salt* | 0.00488 ± 0.000055 | 0.00306 ± 0.000045 |
| *Cad* | 0.00491 ± 0.000049 | 0.00283 ± 0.000034 |
| *Temp* | 0.00479 ± 0.000055 | 0.00290 ± 0.000049 |
| *Spatial* | 0.00515 ± 0.000061 | 0.00312 ± 0.000030 |
| Number of 10kb windows | 7142 | 4004 |

**Table S9. Average diversity (± SE) for the high and low recombination regions in each treatment** **using all sites.**
