# Supplementary material for: Genome-Wide Patterns of Genetic Variation within and among Alternative Selective Regimes
Source: PLoS Genet. 2014 Aug 7;10(8):e1004527. doi: 10.1371/journal.pgen.1004527 (PMC4125100; doi:10.1371/journal.pgen.1004527)
Supplement: Supplemental Information S1 — Evidence of multiple targets of selection underlying differentiation between salt and cadmium environments. (DOCX) [file pgen.1004527.s019.docx]

**Supplementary Information S1 – Evidence of multiple targets of selection underlying differentiation between salt and cadmium environments.**

**Supplementary Information S1A**

On each of the chromosome arms, there are multiple peaks of significant differentiation between populations maintained in salt vs. cadmium environments (Figure 3 and S1).

**Supplementary Information S1B**

**Enrichment of different functional categories across -log(q-value) range**

True targets of selection should be more strongly differentiated and more likely to be in or near genes than sites differentiated by hitchhiking. First, we examined the enrichment of genic SNPs and intergenic SNPs for different -log (q-value) bins [56,57]. If only a few sites in the entire genome are true targets and all other sites become significant due to linkage, we would not expect to be able to see a strong enrichment of the genic sites. Using all the “α-sites”, we calculated the ratio of number of sites located in genic and intergenic region for each -log(q-value) bin. In order to compare the enrichment across different functional categories, we standardized the ratios relative to the mean ratio across the 11 bins. We performed similar enrichment analyses for other functional categories: coding sequences/intron sites, 0-fold sites/4-fold sites in coding region.

There was an enrichment of genic sites relative to intergenic sites in higher significance q-value bins (Figure 3C). Further, we found the enrichment is much stronger when comparing sites located in coding sequence to those located in introns (Figure 3D). We would not expect these patterns if linkage blocks were sufficiently large that sites selected in coding sequence were causing strong differentiation at intron sites. The pattern suggests that the effects of linkage start to decline on a scale smaller than a gene. To assess whether the different initial diversity among categories may produce the enrichment pattern, we repeated the analyses controlling for initial diversity by using only those sites with high initial π (π*_ini_* >0.4, where π*_ini_* = 2*p_ini_*(1 - *p_ini_*) and *p_ini_ =* ½(*p_AS_ + p_AC_*)). The q-values were calculated from the geometric mean value from CMH test between the five *Salt* and five *Cad* populations. The pattern of enrichment remains the same (not shown). Moreover, in the original analysis initial diversity is similar among intergenic, intron and coding sites, suggesting that the enrichment we observed is unlikely due to differences in initial diversity for different site categories.

However, there are limits in our ability to detect enrichment of sites at smaller scales. There is no enrichment of 0-fold sites relative to 4-fold sites for the range of values we consider weakly to strongly significant, i.e., 5 ≤ -log(q-value) < 10 (Figure S2). Contrary to expectation, the ratio of 0-fold sites/4-fold sites is higher among non-significant sites, i.e., -log(q-value) < 4. Purifying selection (in all environments) on some 0-fold sites may actively inhibit differentiation and/or there is differential selection on some 4-fold sites, leading to an enrichment of 0-fold sites relative to 4-fold sites in non-significant classes.

**Supplementary Information S1C**

**Gene annotation and functional clustering**

We tested whether significantly differentiated sites (β-sites) occurred non-randomly with respect to gene function. We used the Gowinda program [59] to identify significantly enriched GO categories. The lower limit of the q-value (after correcting the FDR) for the program is 0.0008 for our data. There are several significantly enriched GO terms (Table S1). Moreover, similar functional categories are identified when gene enrichment tests are performed separately for the two major autosomes. This suggests that the significantly differentiated genes not diverging via hitchhiking to a very small number of selected sites in a same chromosome; rather this enrichment suggests divergence occurs because of selection for functional change.
