# Supplementary material for: Genome-Wide Patterns of Genetic Variation within and among Alternative Selective Regimes
Source: PLoS Genet. 2014 Aug 7;10(8):e1004527. doi: 10.1371/journal.pgen.1004527 (PMC4125100; doi:10.1371/journal.pgen.1004527)
Supplement: Supplemental Information S2 — Evidence of linkage effects contributing to differentiation between salt and cadmium environments. (DOCX) [file pgen.1004527.s020.docx]

**Supplementary Information S2 - Evidence of linkage effects contributing to differentiation between salt and cadmium environments**

**Supplementary Information S2A**

**Clustering of differentiated sites revealed by elevated fraction of β-sites among** α**-sites**

To estimate the level of clustering for the selected sites, we randomly sampled 5000 β-sites and calculated the fraction of β-sites among α-sites within different sizes of windows around each of the selected focal β-sites (excluding the focal sites from the calculation). We report this fraction for a window size averaged across the selected focal sites (Figure S3). To estimate the confidence interval for the average fraction, we bootstrapped across the list of focal β-sites 10 000 times. To obtain a null distribution under the hypothesis of no clustering, we permuted the data by choosing 123 219 α-sites as the pseudo β-sites and used the same focal 5000 sites to calculate the fraction of psuedo β-sites among α-sites around each focal site. We repeated this 1000 times and to obtain 95% confidence intervals for the permuted results. Because false signals could arise due to clustering effects that may occur do to local differences in initial variation, we repeated this analysis using only those α-sites within a certain range of the initial diversity, as measured by π in the Grand Ancestor (0.2 < π*_GA_* ≤ 0.3 or 0.4 < π*_GA_* ≤ 0.5). We found similar patterns using these more restricted data sets.

**Supplementary Information S2B**

***F*_ST_ between environments as function of the distance from selected sites**

Using the five experimental *Cad* populations and the five experimental *Salt* populations, we measured *F*_ST_ around putatively selected sites. As our focal putatively selected sites, we randomly chose 1000 β-sites. For each site, we used non-overlapping 50 base pair windows extending away from the selected site up to 2000 bp in both directions (excluding the significant site in the first window). For each site, *F*_ST_ was calculated as *F*_ST_ = *V*_among environments_/*V*_total_, where

*V*_among environments_ = (*p*_avg__*_Salt_*^2^+*p*_avg__*_Cad_*^2^)/2- ((*p*_avg__*_Salt_* + *p*_avg__*_Cad_*)/2)^2^

*V*_total_ = (*p_Salt_*_1_^2^+ *p_Salt_*_2_^2^+ *p_Salt_*_3_^2^+ *p_Salt_*_4_^2^+ *p_Salt_*_5_^2^ *+p_Cad_*_1_^2^+ *p_Cad_*_2_^2^+ *p_Cad_*_3_^2^+ *p_Cad_*_4_^2^+ *p_Cad_*_5_^2^)/10 - ((*p_Salt_*_1_+ *p_Salt_*_2_+ *p_Salt_*_3_+ *p_Salt_*_4_+ *p_Salt_*_5_ *+p_Cad_*_1_+ *p_Cad_*_2_+ *p_Cad_*_3_+ *p_Cad_*_4_+ *p_Cad_*_5_)/10)^2^

*p*_avg__*_Salt_* = (*p_Salt_*_1_+ *p_Salt_*_2_+ *p_Salt_*_3_+ *p_Salt_*_4_+ *p_Salt_*_5_)/5

and

*p*_avg__*_Cad_* = (*p_Cad_*_1_+ *p_Cad_*_2_+ *p_Cad_*_3_+ *p_Cad_*_4_+ *p_Cad_*_5_)/5

For each window, we calculated the mean *F*_ST_ for all variants within the window, weighted by the total variance among all the populations (*V*_total_). We used only those sites with *V*_total_ >0.05 in the analysis. We calculated the average *F*_ST_ value for the same distance of window across all the focal selected sites. (Figure SI 4a).

As shown in Figure S4A, *F*_ST_ decreases rapidly within 500 to 1000 bp from the focal sites. This indicates that it is unlikely that there are large blocks of strong linkage disequilibrium around the significant sites. We cannot exclude that there are some linkage effects between focal sites and distant sites. Even at the end of 2000 bp distance examined here, the *F*_ST_ is still high, ~0.35. Even at the distance of ~2000bp, ~15% of the sites are q-value significant, different from the proportion of genome-wide significant sites (~6%); this reflects the clustering effect described in Supplementary Information 3. If we randomly select 1000 α-sites and calculate *F*_ST_ using windows that sliding away from these random focal sites, the mean *F*_ST_ for windows of any distance is ~0.25 (Figure S4B). This contrasts with the truly significant sites (Figure S4A) where we see a dramatic drop in *F*_ST_ within the first 1 kb but *F*_ST_ remaining high (~0.3) as far as 20 kb away. This suggests that the selected sites might cluster in certain genomic regions.

**Supplementary Information S2C**

**Linkage disequilibrium within two paired-end reads**

The levels of linkage disequilibrium (LD) within two pair-ends (~250 bp) are estimated as *r*^2^ by the program LDx [60]. Following the manual of the program, we created pileup files, then used samtool and bcftool to transform the file (samtools v. 0.1.16 [52]). The following parameters were used in the LDx program: minimum read depth 10; maximum read depth 100; read from one end of paired end read to the other 500; PHRED quality score cut off: 20; allele frequency cut offs 0.1; minimum intersection depth 5. The *r*^2^ was obtained via the maximum likelihood method for each pair of SNPs. First, we calculated the distance between all two-SNP pairs and used 5 bp windows to bin the reads and calculated the mean *r*^2^ within each window. We then plotted the mean LD against the distance for each bin (Figure S5, blue dots). From these total SNP pairs for LD estimation, we used those SNP pairs where the region between them covered at least one significant site to calculate the mean LD for different distances, weighted by the number of significant sites. The accuracy of LD estimation is lower over longer distances because the intersecting read depth for the two SNPs becomes lower.

Plots of *r*^2^ are shown for each population in Figure S5.

**Supplementary Information S2D**

**Effects of inversions on genetic differentiation**

To examine the effects of inversions on genetic differentiations between cadmium and salt environments, we first estimate the frequency of all the known inversion based on the inversion-specific SNP markers [31]. The inversion frequencies are much smaller than the average allele frequency differentiations across β-sites (Tables S2 and Supplementary Information 3).

To examine whether inversions created strong linked effects among polymorphisms, causing large number of sites differentiated due to linked selection, we compared the proportion of significantly differentiated sites inside and outside the five inversion candidates (Table S4). Average *r*^2^ values inside and outside potentially inverted regions are compared in Table S5.
