# Supplementary material for: Genome-Wide Patterns of Genetic Variation within and among Alternative Selective Regimes
Source: PLoS Genet. 2014 Aug 7;10(8):e1004527. doi: 10.1371/journal.pgen.1004527 (PMC4125100; doi:10.1371/journal.pgen.1004527)
Supplement: Supplemental Information S3 — Calculation of allele frequency correlations between treatments. (DOCX) [file pgen.1004527.s021.docx]

**Supplementary Information S3**

**Calculation of allele frequency correlations between treatments**

Our goal here is to identify sites that might be important in adaptation to either of the salt or cadmium environments and then examine the correlation in allele frequency between treatments. However, positive correlations in allele frequencies can arise because of variation in initial allele frequency (i.e., alleles that are initially at high (low) frequency will tend to be at high (low) frequency in all treatments). Thus, we attempt to obtain a “standardized” correlation by removing the correlation that arises from variation in initial allele frequency. We do this by calculating the correlation observed for putatively selected sites and then subtracting off the correlation observed for control sites.

We first screened for sites that have relatively high coverage (≥15) in both *Ancestral Cad* and *Ancestral Salt* populations. Further, we used only those sites that also had a starting allele frequency for the treatment populations (*p_ini_*=(*p_AC_*+*p_AS_*)/2) of between 0.35 and 0.65 to ensure relatively high initial diversity for both selected and control sites. A Fisher’s exact test (FDR=0.1%) between *AC* and *AS* populations was used to identify the putatively selected sites for this analysis. We use p-value > 0.5 to identify sites to serve as controls. Among the more than 5000 significant sites, we used those separated by at least one control site; when two or more significant sites where adjacent, only one was used. This procedure left us with 1613 significant sites for our analysis. For each significant site, we randomly chose one control site nearby for the control group. For each site, we calculated the mean allele frequency across the five replicates within each treatment. Finally, we calculated the Pearson's product-moment correlation between each pair of treatments for significant sites and then for control sites; we used the difference between them (Diff_Cor = Cor_selected_ - Cor_control_) as a standardized measure of the correlation. To determine the confidence interval for Diff_Cor, we bootstrapped across the 1613 sites (selected and their paired control sites were sampled together) 5000 times. The values for Diff_Cor are given in Table S8.

Amongst all treatment pairs, the most negative value of Diff_Cor is between the *Salt* and *Cad* treatments, suggesting that the most strongly favored alleles in one environment are most strongly disfavored in the other environment. Among all treatment pairs, Diff_Cor is the most positive between *Spatial* and *Temp*, indicating the selected sites behave similarly in the two heterogeneous treatments. The Diff_Cor values for *Salt* with *Temp* or with *Spatial* are negative whereas the Diff_Cor values for *Cad* with *Temp* or with *Spatial* are slightly positive. This seems to suggest that allele frequencies in heterogeneous environments are more strongly governed by cadmium selection than salt selection. However, in the CMH test results (Table 1), the *Temp* treatment has more highly differentiated sites with the *Cad* treatment than with the *Salt* treatment, perhaps implying that selection in the *Temp* treatment is more strongly influenced by salt selection.
