# Supplementary material for: Genome-Wide Patterns of Genetic Variation within and among Alternative Selective Regimes
Source: PLoS Genet. 2014 Aug 7;10(8):e1004527. doi: 10.1371/journal.pgen.1004527 (PMC4125100; doi:10.1371/journal.pgen.1004527)
Supplement: Supplemental Information S4 — Diversity patterns based on the χ-sites outside of possible inversion regions. (DOCX) [file pgen.1004527.s022.docx]

**Supplementary Information S4**

**Diversity patterns based on the χ-sites outside of possible inversion regions**

We re-analyzed the data using only the χ-sites that are located outside the regions where inversions may exist. The resulting diversity patterns (Figure S6) are nosier than the results using all the χ-sites (Figure 5) likely because we excluded ~ 45% of χ-sites in this analysis. Nonetheless the patterns maintain qualitatively similar, suggesting that the inversions are not the main reason causing the diversity difference for sites with different levels of differentiation.
