# Supplementary material for: Genome-Wide Patterns of Genetic Variation within and among Alternative Selective Regimes
Source: PLoS Genet. 2014 Aug 7;10(8):e1004527. doi: 10.1371/journal.pgen.1004527 (PMC4125100; doi:10.1371/journal.pgen.1004527)
Supplement: Supplemental Information S5 — Simulations of experimental populations. (DOCX) [file pgen.1004527.s023.docx]

**Supplementary Information 5 – Simulations of experimental populations**

We simulated 2*N* (2*N* =896) chromosomes for each population. There was one recombination event per pair of chromosomes per generation, occurring with uniform probability across the chromosome. Each chromosome had one neutral site in the middle and four randomly chosen positions for selected loci. To generate initial allele frequencies, we randomly sampled from the empirical data four significantly differentiated sites (β-site) and one non-significant site (non-β-site), identified the derived allele from *Grand Ancestor* and recorded their frequencies in *Ancestral Salt* and *Ancestral Cad* populations. (We screened for 0.05 < π*_ini_* < 0.45 for all five sites and the allele frequency for the non-significant site *p*_neutral_ to be 0< *p*_neutral_ <1 in both ancestral populations. The initial screening of diversity and frequency affected the results quantitatively. But the general patterns are robust, especially for the constant treatments). For each locus, we assumed the allele that was at higher frequency in *AS* than in *AC* was the allele that was favored in the salt environment. To create some random linkage disequilibrium among the five positions, we used the function genPositiveDefMat of “clusterGeneration” package in R [64] to generate covariance matrixes first (with dim=5, covMethod= “unifcorrmat”, rangeVar = c(0,1)). These were converted to correlation matrixes and then were converted back to covariance matrices using the allele frequencies for *AS* and *AC*. (If a correlation matrix could not be converted into a covariance matrix for both *AS* and *AC* then a new correlation matrix was generated.). We then generated haplotypes based on the allele frequencies and the allelic covariances (LD) among the five clusters [65]. To increase the number of selected loci, we split each of the four selected loci into 10, uniformly spanning 0.01 recombination distance. The 10 selected loci all were assigned the same allele frequency and were initially in complete LD with one another. After we created the initial pools of haplotypes from *AC* and *AS*, we made 448 diploid individuals by randomly pairing two haplotypes from these two pools.

We assume selection on each locus is the same and follows the fitness model of *W_AA_* = 1, *W_Aa_* = 1 - ½ *s*, and *W_aa_* = 1-*s* in cadmium and *W_AA_*=1 - *s*, *W_Aa_* = 1 – ½ *s*, and *W_aa_* = 1 in salt. The fitness value for each individual was calculated as the product of the fitness values for all loci ($\prod_{1}^{40} W$). The parents for next generations were sampled with the probability of the relative fitness for each individual.

The two haplotypes from each individual released to the gamete pool for next generation after the random recombination event. For the *Spatial* treatment, there were two sets of haplotypes (2*N*=448) after selection and gamete production from two environments. To produce offspring, each set of haplotypes contributed an equal number of individuals to both environments in the next generation (i.e., soft selection).

We performed 10,000 replicates for each parameter set. For each replicate, we created the initial haplotype pool as described above and then performed five simulations: directional selection on the salt favored allele (representing constant selection in salt environment), directional selection on the cadmium favoured allele (representing constant selection in the cadmium environment), temporally fluctuating selection, spatially variable selection and no selection as a control. Each simulation lasted 42 generations.

Harmonic mean recombination distance between the neutral locus and the selected loci was used to summarize average recombination distance because the effects of linked selection on diversity are usually inversely proportional to recombination distance. The effective recombination distance (*r*) between neutral locus and selected loci is calculated by dividing the harmonic mean distance by the total length of the chromosome. Different physical position maps corresponding to different *r* used the same set of 10,000 initial replicate haplotype pools. Results are shown in Figure S7.
