# Supplementary material for: Genome-Wide Patterns of Genetic Variation within and among Alternative Selective Regimes
Source: PLoS Genet. 2014 Aug 7;10(8):e1004527. doi: 10.1371/journal.pgen.1004527 (PMC4125100; doi:10.1371/journal.pgen.1004527)
Supplement: Supplemental Information S6 — Diversity in regions of high and low recombination. (DOCX) [file pgen.1004527.s024.docx]

**Supplementary Information 6 - Diversity in regions of high and low recombination.**

We divided the genome into low and high recombination rate regions. Based on the estimations in [61], the high region was defined as having a recombination rate greater than 2cM/Mb. We calculated π per 10kb window using the Popoolation program [35,36] and then the mean across low or high recombination windows for each population (Figure S8; Table S9). The number of significantly differentiated sites (β-site) per window is similar between high and low recombination regions (8.3 sites per window for low region and 7.8 sites per window for high region). However, the proportion of β-site to α-sites is higher in low recombination regions than high recombination regions (7.6% vs 5.4%) (Table S9). We also screened the χ-sites for high initial diversity (π*_ini_* > 0.4) and calculated the average π for sites in low recombination and high recombination regions for each treatment (Figure S9).
